# Exploring the Structural Design, Antibacterial Activity, and Molecular Docking of Newly Synthesized Zn(II) Complexes with *NNO*-Donor Carbazate Ligands

**DOI:** 10.3390/molecules30132822

**Published:** 2025-06-30

**Authors:** Claudia C. Gatto, Daniel J. de Siqueira, Eduardo de A. Duarte, Érica C. M. Nascimento, João B. L. Martins, Mariana B. Santiago, Nagela B. S. Silva, Carlos H. G. Martins

**Affiliations:** 1 Laboratory of Inorganic Synthesis and Crystallography, Institute of Chemistry, University of Brasilia, Brasília 70904-970, DF, Brazil; 2 Laboratory of Computational Chemistry, Institute of Chemistry, University of Brasilia, Brasília 70904-970, DF, Brazil; ericamoreno@unb.br (É.C.M.N.); lopes@unb.br (J.B.L.M.); 3 Laboratory of Antimicrobial Testing, Institute of Biomedical Sciences, University of Uberlândia, Campus Umuarama, Uberlândia 38405-320, MG, Brazilcarlos.martins2@ufu.br (C.H.G.M.)

**Keywords:** Zn(II) complexes, carbazate, crystal structures, antibacterial activity, molecular docking

## Abstract

The present work reports the synthesis and structural design of three novel Zn(II) complexes [Zn(L^1^)(CH_3_COO)(H_2_O)] (1), [Zn(L^2^)_2_] (2), and [Zn(L^3^)_2_] (3) with carbazate ligands, 2-acetylpyridine-methylcarbazate (HL^1^), 2-acetylpyridine-ethylcarbazate (HL^2^), and 2-acetylpyridine-benzylcarbazate (HL^3^). All compounds were characterized by spectroscopic methods, and the crystal structures of the complexes were elucidated by single-crystal X-ray. Based on the analysis, distorted square pyramid geometry is suggested for complex (1) and an octahedral geometry is suggested for complexes (2) and (3) with the ligands exhibiting an NNO-donor system. The 3D Hirshfeld surface and the 2D fingerprint plot were used to study the non-covalent interactions in the crystal structures. The in vitro antibacterial investigation of the free ligands and their complexes was performed against different strains of periodontopathogen bacteria. The Zn(II) complexes showed more potent antibacterial activity than the free ligand. Molecular docking studies showed the metal complexes as promising candidates for further therapeutic exploration, particularly in targeting the ATP-binding cassette transporter with peptidase domain of the cariogenic bacteria *S. mutans* (PDB code 5XE9) and the prolyl tripeptidyl aminopeptidase from *P. gingivalis* anaerobic bacteria (PDB code 2EEP) inhibition.

## 1. Introduction

Oral microbiology plays an important role in the body’s overall health. It interacts with the immune system and plays a crucial role in balancing and responding to the immune system [1]. Bacterial infections, such as periodontitis and caries, have been associated with a higher risk of developing different forms of cancer, as indicated in studies on the link between oral diseases and cancer [2]. These infections remain an illness that afflicts thousands of people worldwide, and it is necessary to continue investigating new and more efficient ways to combat these diseases [3]. In this context, it is crucial to develop and investigate new and potent drug molecules to combat bacterial infections.

Schiff bases are compounds of great interest in coordination and bioinorganic chemistry due to their chelating potential and varied applications [4,5,6,7]. There is a great diversity of metal complexes with Schiff bases in their composition; some of their properties can be mentioned, such as their catalytic capacity, as being used in the oxidation of alcohols and as chemical sensors for detecting transition metals, such as copper, silver, chromium, and others [8,9]. Furthermore, the properties of great general interest have pharmacological and biological applications. Studies showed that metal complexes with Schiff base ligands can present promising biological activities such as antimicrobial, antibacterial, and anticancer [10,11,12,13,14].

Carbazate ligands are Schiff bases with the potential to coordinate with different transition metals, forming stable complexes with varied pharmacological applications, including their cytotoxic potential against various cancer cells [15,16,17]. Metal complexes with carbazate ligands were also successfully studied in several areas of medicine and biology, from research related to Chagas disease, as fluorescent markers in cancer cells for evaluation by electron microscopy, and against different types of bacteria [18,19,20,21,22].

Zinc complexes with Schiff base ligands are interesting due to the possible geometries they can adopt and promising pharmacological activities [23,24,25,26,27,28]. In previous work, metal complexes with carbazate ligands were synthesized and showed better antimicrobial activity when compared to free ligands. Synthesized complexes of Ni(II), Cu(II), and Zn(II) showed potent antimicrobial and antitubercular activities, with promising activity when compared to the drug reference [22,29].

In this paper, we describe the synthesis of three Zn(II) complexes derived from the ligands 2-acetylpyridine-methylcarbazate (HL^1^), 2-acetylpyridine-ethylcarbazate (HL^2^), and 2-acetylpyridine-benzylcarbazate (HL^3^). FT-IR, electronic spectroscopy, mass spectrometry, ^1^H NMR, and CHN elemental analysis characterized the newly synthesized compounds. The crystal structures of the complexes were elucidated by X-ray diffraction, and the Hirshfeld surface was studied. Biological tests were also carried out to verify the possible antimicrobial activities of the compounds. Additionally, molecular docking was carried out to explore the binding affinity of the carbazate ligand and its Zn(II) complexes with two different bioreceptors, the ATP-binding cassette transporter peptidase domain (PEP) [30] of the cariogenic bacteria *S. mutans* and the prolyl tripeptidyl aminopeptidase (PTP39) from *P. gingivalis* anaerobic bacteria [31].

## 2. Results and Discussion

**Scheme 1:**
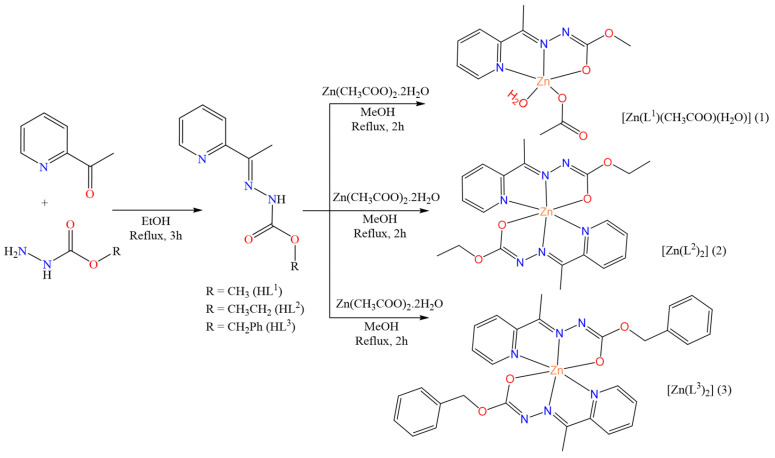
Synthesis of the Schiff base (HL^1^, HL^2^, and HL^3^) and their Zn(II) complexes (1–3).

The carbazate ligands HL^1^, HL^2^, and HL^3^ were synthesized by the condensation reaction of 2-acetylpyridine with methylcarbazate, ethylcarbazate, and benzylcarbazate, respectively. The reactions were carried out in ethanol with 3 h of reflux to ensure the formation of the azomethine group. Their Zn(II) complexes were obtained from the reactions between the carbazate ligands and Zn(II) acetate, as seen in Scheme 1. Spectroscopic analyses indicated the formation of the complexes where Zn(II) atoms were coordinated to the ligand deprotonated by the *NNO*-donor system. Physicochemical and spectroscopic methods characterized the compounds. The crystal structures of the complexes (1–3) were established by single-crystal X-ray diffraction.

### 2.1. Structural Analyses

**Figure 1:**
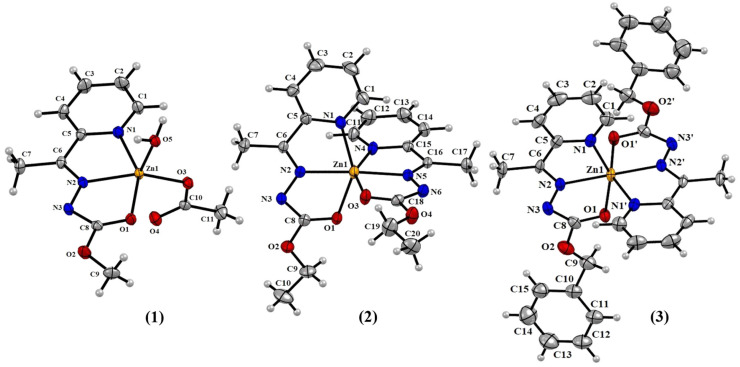
Molecular structure of the Zn(II) complexes (1–3). Thermal ellipsoids with 30% of probability. Symmetry operation (′): −x + 1, y, −z + 1/2.

The crystal structures of complexes (1–3) were elucidated by single-crystal X-ray diffraction, and the ORTEP representations are in Figure 1. The crystal structure determination of complex (1) with HL^1^ revealed the metal center coordinated to a deprotonated and tridentate ligand molecule, through the nitrogen atoms of the pyridine ring (N1), azomethine nitrogen (N2), and enol oxygen (O1). The Zn(II) atom adopted a distorted square pyramidal geometry, with a water molecule occupying the apical position. The basal plane was defined by a ligand molecule and an acetate ion, exhibiting a torsion angle of 18.12°. The bond distance Zn1–O1 of 2.137 (15) Å was longer than Zn1–O3 of 1.957 (16) Å and Zn1–O5 of 2.009 (19) Å and can be explained by the rigidity of the ligand molecule compared to the water molecule and the acetate ion. The Addison parameter of 0.0558 was calculated with the angles N1–Zn1–O1 of 151.55° and N2–Zn1–O3 of 148.20°, giving a near-perfect square-based pyramid for the metal ion [32].

Complexes (2) and (3) with HL^2^ and HL^3^, respectively, showed the metal ion hexacoordinatedly inserted into a distorted octahedral geometry, coordinated with two ligand molecules in an N_4_O_2_ coordination mode of the ligand molecules. The Schiff base showed similar coordination to the two previous examples by the *NNO*-donor system (Figure 1). In complex (2), the carbazate ligand molecules were deprotonated and in their enol form, with an increase in the bond length C8–O1 of 1.235 (4) Å compared to the 1.204 (2) Å bond length in the free ligand HL^2^. However, complex (2) showed shorter bond lengths C8-N3 of 1.334 (5) Å compared to the free ligand of 1.349 (2) Å, indicative of the delocalization of electrons through the carbazate function when coordinated with the metal center. This same behavior was observed for complex (3), with bond lengths C8–O1 and C8–N3 of 1.248 (4) and 1.336 (5) Å, respectively. The bond lengths were consistent with those found in the literature for other zinc complexes [33,34]. The distorted octahedral geometry was evident from the significant variation in bond angles, with cis angles ranging from 73.54 (2)° to 99.26 (2)° and trans angles between 148.72 (2)° and 173.29 (3)°, as observed in similar reported Zn(II) complexes [35,36]. Table 1 shows selected bond distances and angles for the complexes (1–3).

The crystal structure of complex (1) presents a series of hydrogen bonds among the hydrogen atoms of the coordinated water molecule, the oxygen atom from the acetate ion, and the deprotonated nitrogen atom N3. These interactions formed a one-dimension network with a distance of 1.94 (3) Å and an angle of 160 (3)° for the O5–H5B∙∙∙O3 interaction (symmetry operation −x + 1, −y + 1, −z + 2) and a distance of 1.82 (3) Å and an angle of 174 (3)° for the O5–H5B∙∙∙O3 interaction (symmetry operation −x + 1, −y + 1, −z + 1) (Figure S1 in the Supplementary Materials).

### 2.2. Hirshfeld Surface

**Figure 2:**
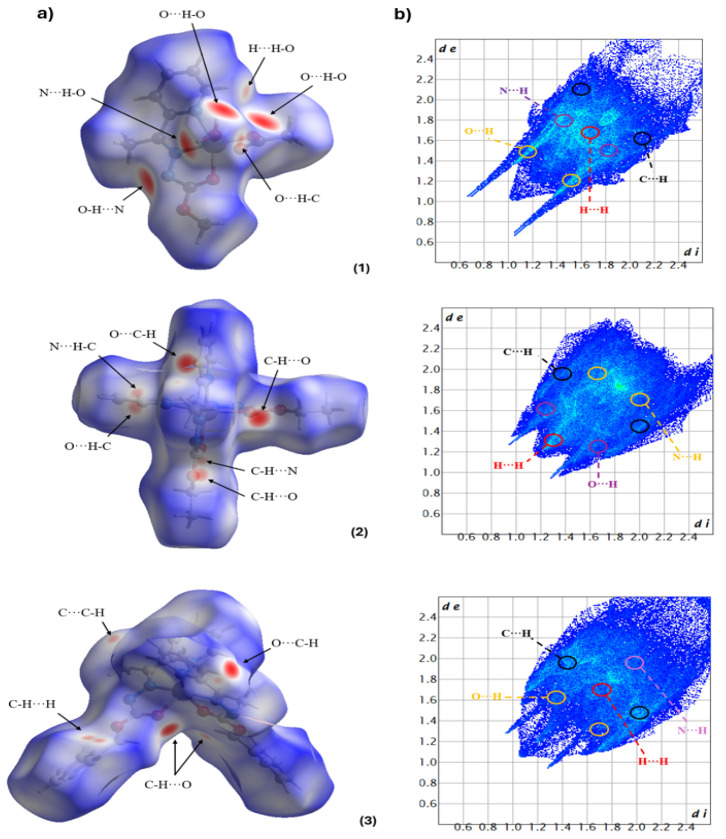
Hirshfeld surface analysis mapped with d_norm_ (**a**) and full two-dimensional fingerprint plot (**b**) showing diverse non-covalent interactions of complexes (1–3).

The Hirshfeld surface (HS) was used to analyze and quantify the non-covalent interactions that make up the crystal lattice [37,38]. HS and fingerprint graph calculations were performed using data obtained by X-ray diffraction and the CrystalExplorer 21.5 program [39]. The d_norm_ function in HS is obtained by combining the functions d_i_ and d_e_, where d_i_ is a function of the distance between the surface and the closest internal atom, and d_e_ is a function of the distance between the surface and the closest external atom. The obtained function d_norm_ showed red regions representing contacts smaller than the sum of the van der Waals radii of the involved atoms, the blue regions indicating contacts larger than the sum of the van der Waals radii, and the white regions were close to the sum of the van der Waals radii. HS analyses showed non-covalent interactions for all complexes. The more intense red regions were observed in complex (1) due to the intermolecular hydrogen bonds between O–H∙∙∙O of the acetate ion and water molecule coordinated to the metal center with bond distances of 1.82 (3) and 1.94 (3) Å. Similar to Ni(II) complexes with carbazate ligands, nonclassical interactions such as C–H∙∙∙O and C–H∙∙∙H interactions were also observed in the structures of (1–3) [22]. No interactions were found for the phenyl ring in (3), resulting in increased vibration and disorder of the aromatic ring. Figure 2 shows the HS and full fingerprint of complexes (1–3).

**Figure 3:**
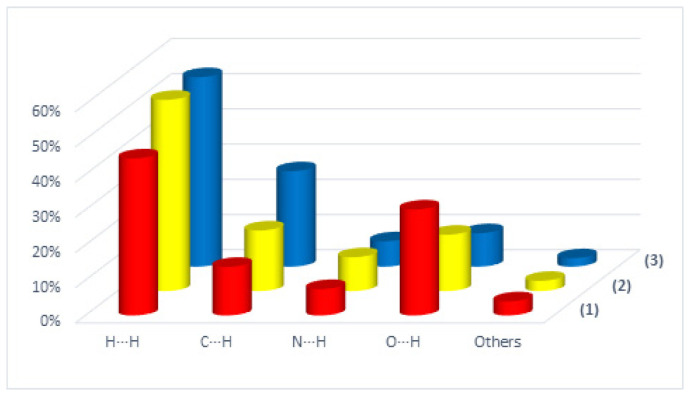
Contribution of some intermolecular contacts for the HS of complexes (1–3).

In all structures, there was a large contribution from the H∙∙∙H and C∙∙∙H contacts, with a variation between 54.3 and 44.6% of the H∙∙∙H contribution and a variation between 27.1 and 13.8% of the C∙∙∙H contribution. A significant contribution to the crystal packing for complex (1) was observed due to the O∙∙∙H contacts of 30.1% compared with 16 and 9.5% for complexes (2) and (3), respectively. Figure 3 shows the contributions of some contacts for complexes (1–3), obtained from the decomposition of the fingerprint plots of the complexes (Supplementary Materials Figures S2–S4), the fingerprint plots of the complexes showing the percentages of contacts contributing to the total Hirshfeld surface area.

### 2.3. Infrared Spectra

The FT-IR spectra of the Schiff bases and their complexes (1–3) were obtained using the KBr pellet method (Supplementary Materials Figures S5–S10) and compared to verify the occurrence of coordination of the ligand to the metal ion. In the spectra of the ligands, the main bands observed were around 3222–3262, 1702–1716, 1618–1623, 1567–1580, 1152–1159, and 747–783 cm^−1^, which were assigned to ν(N–H), ν(C=O), ν(C=N), ν(C=N_py_), ν(N–N), and δ(py), respectively. In the spectra of complexes (1–3), it was possible to observe the disappearance of the band ν(N–H), which suggested the deprotonation of the ligand. In complexes (2–3), it was possible to notice the absence of bands attributed to ν(C=O) in the spectra, which indicated that the ligand was coordinated to the zinc atom in its enol form. In the complex (1) spectrum, a band ν(C=O) was observed at 1696 cm^−1^, which can be attributed to the acetate ion coordinated to the zinc atom since the ligand was deprotonated and in enol tautomer. A shift in the stretching frequencies of the C=N bonds of pyridine and azomethine was observed in the spectra of complexes (1–3), with the stretching ν(C=N_py_) originally between 1567 and 1580 cm^−1^ shifted to 1532–1563 cm^−1^ and in the stretching ν(C=N) of azomethine a change was observed from 1618–1623 cm^−1^ to 1566–1584 cm^−1^; this shift in the stretching of the C=N bonds suggested the coordination of the ligand by nitrogen atoms. Infrared spectroscopy indicated that the ligand was coordinated with the zinc atoms by the *NNO*-donor system in the three complexes; similar results were observed in previous works [40,41,42,43].

### 2.4. Electronic Spectra

The absorption spectra in the ultraviolet region of the ligands HL^1-3^ and complexes (1–3) were obtained in MeOH, DMF, and DMSO (2 × 10^−5^ M) to evaluate the impact of the solvent on the electronic spectrum (Supplementary Materials Figures S11–S13 and Table S1). In the spectra of the three ligands, it was possible to observe that absorption occurred in the region between 284 and 288 nm, depending on the solvent. This absorption band can be attributed to transitions π→π* of the azomethine group and pyridine. The bands in the region between 347 and 368 nm were characteristic of the n→π* transitions, originating from the non-bonding electrons of the heteroatoms present in the carbazate fraction [10]. When we evaluated the spectrum of complexes (1–3) and compared it with that of the ligands, a bathochromic shift in the absorption bands was observed. The π→π* absorption band shifted from 284–288 nm to 285–295 nm in the complexes. The bathochromic shift was caused by the decrease in energy between the HOMO and LUMO orbitals; this decrease in energy caused an electron from the HOMO orbital to be promoted to a LUMO orbital [44]. More evidence of ligand coordination was the appearance of the intense band in the 347–383 nm region. This band referred to the transfer of a metal–ligand charge between the *NO* sites of the ligand and the metal. Due to the zinc atom being a metal with electronic configuration d^10^, the d–d transitions were not observed.

### 2.5. H NMR Spectra

The ^1^H nuclear magnetic resonance spectra of complexes (1–3) and the free ligands were obtained in DMSO-d_6_ (Supplementary Materials Figures S14–S19). In the spectrum of the HL^1^ and complex (1), peaks relating to the aromatic hydrogen atoms of the pyridine ring were observed in the region between 7.35 and 8.57 ppm. The singlets at 3.38 and 3.60 ppm can be attributed to the CH_3_ group linked to the oxygen atom of the ester. The signal from CH_3_ bound to azomethine appeared as a singlet at 2.30 and 2.54 ppm, in HL^1^ and complex (1), respectively. The peak at 10.3 ppm in the HL^1^ spectrum can be attributed to NH hydrogen; the absence of this peak in the spectrum of the complex showed that the ligand was deprotonated when coordinated. In the spectrum of complex (1), the appearance of a singlet at 3.69 ppm was due to the CH_3_ group of the acetate coordinated to the metal. The aromatic hydrogen atoms of the HL^2^ ligand and complex (2) can be assigned to the peaks between 7.36 and 8.58 ppm in the spectra. The triplet and quartet at 1.28 and 4.21 ppm in the HL^2^ spectrum and at 1.12 and 4.03 ppm in the spectrum of complex (2) referred to the CH_3_CH_2_ group. The CH_3_ bonded to azomethine peaks as a singlet at 2.31 and 2.54 ppm in the spectra of HL^2^ and complex (2), respectively. The singlet referring to NH at 10.31 ppm was also not observed in the spectrum of the complex, which confirmed that the ligand molecules were deprotonated. For the spectra of complex (3) and HL^3^, we observed a range between 7.22 and 8.57 ppm, where the peaks of the aromatic hydrogen atoms of the pyridine and the aromatic ring of the carbazate portion were found. The CH_2_ bonded to the aromatic ring, and oxygen presented its signal at a greater chemical shift due to the deshielding caused by the aromatic ring and oxygen, 5.23 and 5.08 ppm for HL^3^ and complex (3), respectively. The singlets at 2.31 and 2.51 ppm denoted CH_3_ bonded to the azomethine group. In solution, the ^1^H NMR spectra suggested that the ligands were deprotonated with coordination to the zinc atoms due to the absence of the NH hydrogen peak at 10.44 ppm, as in the spectra of the free ligands.

### 2.6. Antibacterial Activity

The free carbazate ligands and their Zn(II) complexes (1–3) had their antibacterial potential evaluated against six cariogenic bacteria strains, *S. mutans*, *S. sobrinus*, *S. oralis*, *S. sanguinis*, *S. salivarius*, and *L. paracasei*, and five anaerobic bacteria strains of the periodontal infections, *A. naeslundii*, *P. anaerobius*, *V. parvula*, *P. gingivalis*, and *F. nucleatum*. The drug control used to compare the antibacterial activity was chlorhexidine. Minimum inhibitory concentration (MIC) values were obtained for all compounds, which indicated the minimum concentration for the inhibition of bacterial growth to occur, and are presented in Table 2.

Among the ligands, the HL^3^ ligand showed superior antibacterial activity to the others, requiring a more than ten times lower concentration to inhibit bacterial growth. It was also possible to observe a reduction in the MIC when the ligands were complexed with the metal, which indicated an improvement in antibacterial activity with the presence of the metal. In general, the HL^3^ carbazate and its complex (3) showed better activity against the tested cariogenic and anaerobic mycobacteria strains, except against *P. gingivalis*, against which complex (2) presented less MIC (50 μg mL^−1^). The most promising compound with the best bacterial activity was complex (3), where the best results were against the *S. mutans*, *S. sobrinus*, and *P. anaerobius* bacteria strains with an MIC of 12.5 μg mL^−1^. It is interesting to note that the ligand with the largest organic group (–CH_2_Ph) in its chain, as well as its complex (3), produced a greater interaction and antibacterial activity. A comparative analysis of Zn(II) and Ni(II) complexes with carbazate ligands revealed that Zn(II) complexes exhibited more promising antibacterial activity against various bacteria [22]. The antimycobacterial activity observed was similar to Zn(II) complexes with other Schiff bases [45,46,47,48]. Zinc demonstrated significant antibacterial effects, emerging as a promising candidate for periodontal applications. Recent studies emphasized the potential of zinc and its compounds to combat periodontal pathogens, highlighting the importance of further investigating its properties [49,50].

### 2.7. Molecular Docking Study

#### 2.7.1. Docking of PEP–Ligands Systems

**Figure 4:**
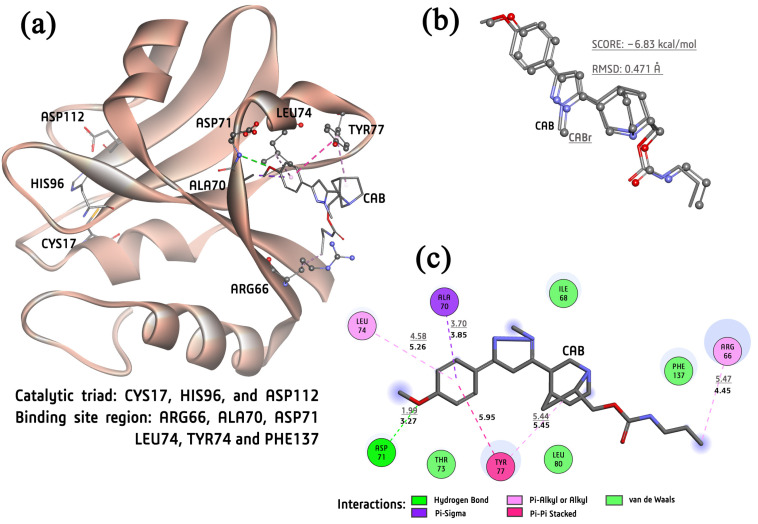
A 3D representation of the PEP active site and main residues interacting with the CAR inhibitor, PDB code 5XE9. (**a**) Catalytic triad and binding site region; (**b**) redocking superposition CAB (tube) and CABr (ball and stick) poses; (**c**) 2D representation of main interactions in the complexes PEP–CAB (distances/Å-written in black) and PEP–CABr (distances/Å-written in gray).

The PEP is a peptidase of the family of cysteine proteases with high specificity. This enzyme is part of the proteome of the quorum-sensing system of the bacteria *S. mutans* [30]. The catalytic triad of the PEP enzyme is in the residues Cys17, His96, and Asp112; nevertheless, the hydrophobic pocket of the enzyme is also the binding site of the *N*-terminal helix (Figure 4a) and the majority of ligand CAB’s five hydrophobic interactions with residues Ala70, Tyr77, Leu74, and Arg66 and just a hydrogen bond with residue Asp71.

The docking study of the interactions between the PEP enzyme and the ligands synthesized in our group showed that complex (3) and ligand HL^3^ were the best inhibitor candidates (Table 3). Both molecules mimicked the same profile of interactions as the ligand CAB, performing the majority of several hydrophobic interactions and a few hydrogen bonds. Complex (3) interacted with the hydrogen atoms of the NH_2_^+^ Arg66 residue, forming a strong hydrogen bond with its two oxygen atoms at 1.79 and 2.33 Å.

**Figure 5:**
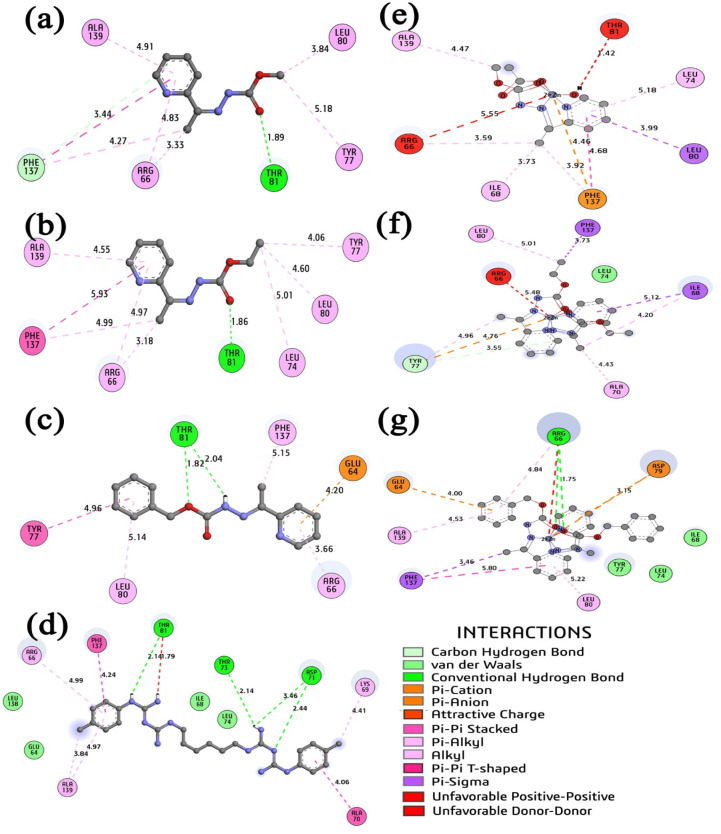
Docking best pose (score) of complexes formed between the PEP active site residues and the three carbazate ligands and their Zn(II) complexes (1–3) and chlorhexidine. (**a**) A 2D representation of main interactions (distance in Å) of the PEP–HL^1^ complex; (**b**) 2D representation of main interactions (distance in Å) of the PEP–HL^2^ complex; (**c**) 2D representation of main interactions (distance in Å) of the PEP–HL^3^ complex; (**d**) 2D representation of main interactions (distance in Å) of the PEP–chlorhexidine complex; (**e**) 2D representation of main interactions (distance in Å) of the PEP–(1) complex; (**f**) 2D representation of main interactions (distance in Å) of the PEP–(2) complex; (**g**) 2D representation of main interactions (distance in Å) of the PEP–(3) complex.

Comparing the order of inhibition in the experimental assays presented in Table 3 for the *S. mutans* bacteria with the predicted order of inhibition in our docking study revealed a similar trend, with the same order of magnitude (in micromolar) for theoretical Ki and experimental values in almost all cases. Ligand HL^3^ and its complex (3) presented strong similarity in the type of interactions performed with the PEP enzyme binding site, as shown in Figure 5c,g. The main difference was the intermolecular interaction between those molecules and the residue Arg66; complex (3), in interacting with charged Arg66 residue, made two hydrogen bonds, a pi-alkyl interaction (at 4.84 Å), and an unfavorable interaction checked in the repulsion due the proximity of the Zn(II) ion and the charged atom N^+^ of the Arg66. This unfavorable interaction may have been responsible for the same magnitude of the energy of binding predicted for complex (3) and its ligand. In contrast, a strong binding energy was expected for complex (3).

#### 2.7.2. Docking of PTP-39-Ligands Systems

**Figure 6:**
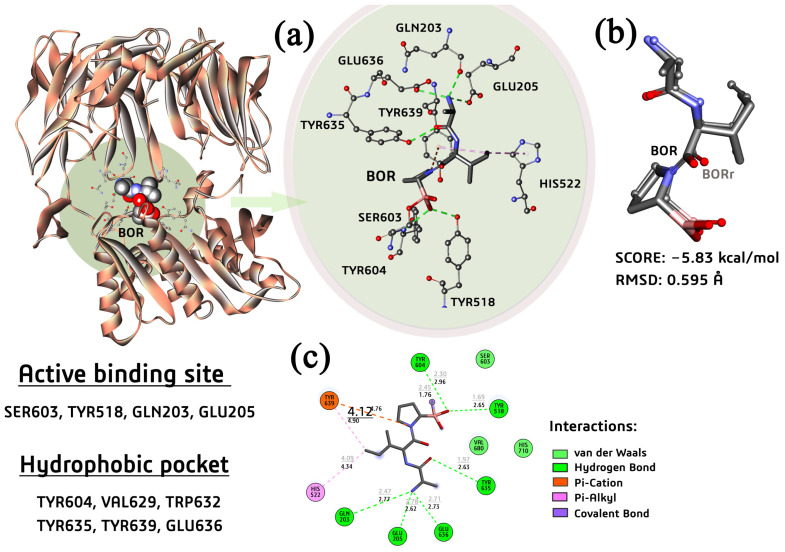
PTP39 active site and main residues interacting with BOR inhibitor, PDB code 2EEP. (**a**) A 3D representation of the main active site residues interacting with BOR inhibitor, PDB code 2EEP; (**b**) redocking superposition BOR (tube) and BORr (ball and stick) poses; (**c**) 2D representation of main interactions in the complex PTP39–BOR (distances/Å-written in black) and PTP–BORr (distances/Å-written in gray).

The Gram-negative anaerobe bacteria *P. gingivalis* presented in their complex proteomic bulk a prolyl tripeptidyl aminopeptidase (PTP39), an enzyme with 659 residues in its structure. The active binding site of the PTP39 is located in a hydrophobic cavity, where the catalytic residue Ser603 and Tyr518 can form strong interactions as a covalent bond and short-range hydrogen bonds with some types of small molecules (Figure 6).

Our docking study revealed that ligand HL^3^ and complex (2) presented a similar molecular profile of interactions as the ligand BOR with key residues of the active site of the PTP39. The interactions with the catalytic residue Ser603 indicated if the small molecule could inhibit the enzyme. The number of hydrophobic interactions performed in the complex formation between the PTP39-ligand was an additional condition for a good inhibitory profile. Our docking study predicted a similar trend in the binding energy (Table 4) as the experimental assay (Table 2), with a discrepancy in the position between the HL^3^ and complexes (2) and (3). Ligand HL^3^ emulated the same interactions in the active catalytic site as the BOR ligand, but, in the opposite way, complexes (2) and (3) presented similarities in the hydrophobic interactions in the hydrophobic pocket of the PTP39.

**Figure 7:**
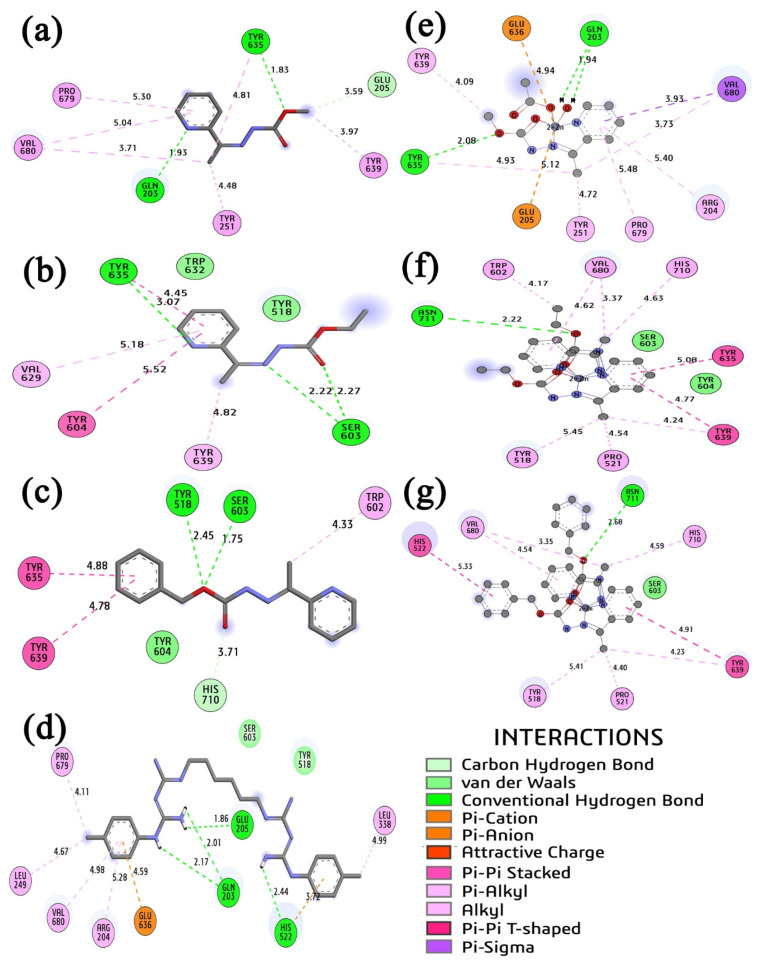
Docking best pose (score) of complexes formed among the PTP39 active site residues and the three carbazate ligands and their Zn(II) complexes (1–3) and chlorhexidine. (**a**) A 2D representation of main interactions (distance in Å) of the PTP39–HL^1^ complex; (**b**) 2D representation of main interactions (distance in Å) of the PTP39–HL^2^ complex; (**c**) 2D representation of main interactions (distance in Å) of the PTP39–HL^3^ complex; (**d**) 2D representation of main interactions (distance in Å) of the PTP39–chlorhexidine complex; (**e**) 2D representation of main interactions (distance in Å) of the PTP39–(1) complex; (**f**) 2D representation of main interactions (distance in Å) of the PTP39–(2) complex; (**g**) 2D representation of main interactions (distance in Å) of the PTP39–(3) complex.

As shown in Table 4 and Figure 7c,f,g, the ligand HL^3^ presented a strong hydrogen bond interaction (1.75 Å) with a similar distance to the covalent bond performed with the boron ligand BOR (1.76 Å) determined by crystallographic X-ray. The complex (2) design had the same hydrophobic interactions as BOR while complexed with the PTP39 enzyme. On the other hand, complex (3) and chlorhexidine also presented an intermolecular interaction with the catalytic His522 residue and other residues of the active binding site, forming the most stable complexes in the in silico study.

## 3. Materials and Methods

### 3.1. Materials, Methods, and Instruments

The solvents and reagents were acquired from Merck (São Paulo, Brazil) and used directly after purchase without additional purification. Elemental analysis of CHN was performed with a Perkin Elmer/Series II 2400 analyzer (Perkin Elmer, Shelton, CT, USA). The FT-IR spectra of the compounds were obtained from KBr pellets, using a FT-IR Varian 640 spectrometer (Agilent Technologies, Santa Clara, CA, USA) in the range of 4000–400 cm^−1^. The ^1^H nuclear magnetic resonance spectra were collected on a Varian Mercury Plus (300 MHz), with TMS as internal reference and DMSO-d_6_ as solvent (UC Davis RMN Facility, Sacramento, CA, USA). UV-Vis-NIR Varian Cary 5000 spectrophotometer was used (Agilent Technologies, CA, USA), and the concentration used for all analyses was 2 × 10^−5^ M in methanol (MeOH), dimethylformamide (DMF), and dimethylsulfoxide (DMSO).

### 3.2. Synthesis of Carbazate Ligands (HL-HL^3^)

The mixture of methylcarbazate (1 mmol, 91 mg) and 2-acetylpyridine (1 mmol, 121 mg) with 15 mL of ethanol was refluxed and heated for 3 h. Colorless crystals were obtained after slow evaporation of the mother solution at room temperature. The procedure was carried out similarly for HL^2^ and HL^3^, changing methylcarbazate with ethylcarbazate (1 mmol, 105 mg) and benzylcarbazate (1 mmol, 168 mg), respectively.

HL^1^: Yield: 96%. Anal. calcd for C_9_H_11_N_3_O_2_ (%): C, 55.95; H, 5.74; N, 21.75 found (%): C, 55.82; H, 5.91; N, 21.73. Selected IR bands (KBr, ν/cm^−1^): ν(N–H) 3243, ν(C=O) 1702, ν(C=N) 1623, ν(C=Npy) 1567, ν(N–N) 1152, δ(py) 783. ^1^H NMR (DMSO-d_6_ δ, ppm): 2.30 (s, 3H, CH3); 3.38 (s, 3H, CH3); 7.37 (t, 1H, Ar); 7.82 (t, 1H, Ar); 7.99 (d, 1H, Ar); 8.57 (d, 1H, Ar); and 10.37 (s, 1H, N–H). UV-vis (MeOH): λmax = 284 nm and 347 nm. UV-vis (DMF): λmax = 287 nm and 356 nm. UV-vis (DMSO): λmax = 288 nm and 377 nm.

HL^2^: Yield: 92%. Anal. calcd for C_10_H_13_N_3_O_2_ (%): C, 57.96; H, 6.32; N, 20.28 found (%): C, 57.74; H, 6.27; N, 20.17. Selected IR bands (KBr, ν/cm^−1^): ν (N–H) 3262, ν(C=O) 1711, ν(C=N) 1620, ν(C=Npy) 1580, ν(N–N) 1159, δ(py) 747. ^1^H NMR (DMSO-d_6_ δ, ppm): 1.28 (s, 3H, CH3); 2.31 (s, 3H, CH3); 4.21 (q, 2H, CH2); 7.38 (t, 1H, Ar); 7.82 (t, 1H, Ar); 8.00 (d, 1H, Ar); 8.58 (d, 1H, Ar); and 10.31 (s, 1H, N–H). UV-vis (MeOH): λmax = 285 nm and 357 nm. UV-vis (DMF): λmax = 287 nm and 367 nm. UV-vis (DMSO): λmax = 288 nm and 377 nm.

HL^3^: Yield: 91%. Anal. calcd for C_15_H_15_N_3_O_2_ (%): C, 66.90; H, 5.61; N, 15.60 found (%): C, 67.03; H, 5.55; N, 15.97. Selected IR bands (KBr, ν/cm^−1^): ν(N–H) 3222, ν(C=O) 1716, ν(C=N) 1618, ν(C=Npy) 1580, ν(N–N) 1155, δ(py) 745. ^1^H NMR (DMSO-d_6_ δ, ppm): 2.31 (s, 3H, CH3); 5.23 (s, 2H, CH2); 7.40–8.57 (m, 9H, Ar) and 10.44 (s, 1H, N–H). UV-vis (MeOH): λmax = 285 nm and 355 nm. UV-vis (DMF): λmax = 288 nm and 368 nm. UV-vis (DMSO): λmax = 288 nm and 383 nm.

### 3.3. Synthesis of the Complexes [Zn(L^1^)(CH_3_COO)(H_2_O)] (1), [Zn(L^2^)_2_] (2), and [Zn(L^3^)_2_] (3)

For the synthesis of complex (1), Zn(CH_3_COO)_2_·2H_2_O (0.1 mmol, 22 mg) was dissolved in 5 mL of methanol and added to a solution of 5 mL of methanol with HL^1^ (0.1 mmol, 20 mg). The mixture was refluxed and heated for 2 h. Yellow crystals suitable for X-ray diffraction were obtained directly from the mother liquor upon standing the solution at room temperature for several days. The same procedure was performed for the synthesis of complexes (2) and (3), replacing HL^1^ with HL^2^ (0.1 mmol, 21 mg) or HL^3^ (0.1 mmol, 27 mg), respectively.

(1): Yield: 61%. Anal. calcd for C_11_H_15_N_3_O_5_Zn (%): C, 39.48; H, 4.52; N, 12.56 found (%): C, 39.26; H, 4.69; N, 12.35. Selected IR bands (KBr, ν/cm^−1^): ν(O–H) 3412, ν(C=O) 1696, ν(C=N) 1584, ν(C=Npy) 1563, ν(C–O) 1293, ν(N–N) 1177, δ(py) 783. ^1^H NMR (DMSO-d_6_ δ, ppm): 2.54 (s, 3H, CH3); 3.60 (s, 3H, CH3); 3.69 (s, 3H, CH3); 7.35 (t, 1H, Ar); 7.80 (d, 1H, Ar); 7.99 (t, 1H, Ar) and 8.46 (s, 1H, Ar). UV-vis (MeOH): λmax = 285 nm and 349 nm. UV-vis (DMF): λmax = 290 nm and 361 nm. UV-vis (DMSO): λmax = 295 nm and 363 nm.

(2): Yield: 56%. Anal. calcd for C_20_H_24_N_6_O_4_Zn (%): C, 50.27; H, 5.06; N, 17.50 found (%): C, 50.27; H, 5.23; N, 17.42. Selected IR bands (KBr, ν/cm^−1^): ν(O–H) 3413, ν(C=N) 1568, ν(C= Npy) 1537, ν(C–O) 1277, ν(N–N) 1158, δ(py) 774. ^1^H NMR (DMSO-d_6_ δ, ppm): 1.12 (s, 6H, CH3); 2.54 (s, 6H, CH3); 4.03 (q, 4H, CH2); 7.36 (t, 2H, Ar); 7.81 (d, 2H, Ar); 7.98 (t, 2H, Ar) and 8.19 (d, 2H, Ar). UV-vis (MeOH): λmax = 286 nm and 348 nm. UV-vis (DMF): λmax = 290 nm and 362 nm. UV-vis (DMSO): λmax = 294 nm and 363 nm.

(3): Yield: 51%. Anal. calcd for C_30_H_28_N_6_O_4_Zn (%): C, 59.86; H, 4.69; N, 13.96 found (%): C, 59.79; H, 4.75; N, 13.86. Selected IR bands (KBr, ν/cm^−1^): ν(O–H) 3430, ν(C=N) 1566, ν(C= Npy) 1532, ν(C–O) 1280, ν(N–N) 1170, δ(py) 775. ^1^H NMR (DMSO-d_6_ δ, ppm): 2.51 (s, 6H, CH3); 5.08 (q, 4H, CH2) and 7.40–8.57 (m, 18H, Air). UV-vis (MeOH): λmax = 285 nm and 347 nm. UV-vis (DMF): λmax = 290 nm and 361 nm. UV-vis (DMSO): λmax = 292 nm and 361 nm.

### 3.4. Crystal Structure Determination

Data collection of the compounds was determined by single-crystal X-ray diffraction and performed on a Bruker CCD SMART APEX II diffractometer, in which a graphite monochromator was used and had a Mo-Kα (0.71073 Å) source at 293 K. The structures were solved using the SHELXT program [51], and the positions of non-hydrogen atoms were determined using subsequent Fourier-difference map analyses. Refinement was carried out using the SHELXL [52] with minimization of least squares. The Olex2 program [53] was used for both the solution and refinement of the structures. Molecular graphics were generated with Olex2 and MERCURY 2024.2.0 software [53,54]. Crystal data, experimental details, and refinement results are summarized in Supplementary Materials Table S2.

### 3.5. Computational Details

The Hirshfeld surface (HS) and 2D fingerprint calculations were performed using the software CrystalExplorer 21.5 [39], using the Crystallographic Information Files (CIFs) obtained by single-crystal X-ray. The 3D d_norm_ surfaces (normalized contact distances) of the compounds were mapped using a scale of −0.7136 (red) and 1.4439 (blue). Combining the functions d_i_ and d_e_ resulted in the 2D fingerprint plot; this function was used to qualitatively and quantitatively evaluate the intermolecular interactions of the compounds.

### 3.6. Biological Activity

#### 3.6.1. Bacteria Used and Cultivation Conditions

The bacteria used in the study were sourced from the American Type Culture Collection (ATCC) and are part of the culture collection at the Laboratory of Antimicrobial Testing (LEA) of the Federal University of Uberlândia, State of Minas Gerais, Brazil. They are maintained and stored in ultra-freezers at −80 °C. These included six cariogenic strains, *S. sanguinis* (ATCC 10556), *S. oralis* (ATCC 55229), *S. mutans* (ATCC 25175), *S. sobrinus* (ATCC 33478), *S. salivarius* (ATCC 25975), and *L. paracasei* (ATCC 11578), cultured in Brain Heart Infusion broth (BHI, Sigma, St. Louis, MO, USA) or BHI agar (Sigma) supplemented with 5% defibrinated horse blood. Incubation was carried out in a bacteriological incubator under an atmosphere containing 10% CO_2_ and 60–80% humidity at 37 °C for 24 h. Additionally, *S. salivarius* (ATCC 25975) was cultured aerobically for 24 h at 37 °C in BHI broth or BHI agar supplemented with 5% defibrinated horse blood. For the five periodontopathogenic strains, including *V. parvula* (ATCC 17745), *P. anaerobius* (ATCC 27337), *F. nucleatum* (ATCC 19053), *A. naeslundii* (ATCC 19039), and *P. gingivalis* (ATCC 49417), they were cultured in Brucella broth (Sigma Chemical Co Analytical, St Louis, MO, USA) supplemented with hemin (5 μg/mL) and menadione (1 μg/mL) or Brucella agar (Sigma Chemical Co Analytical, St Louis, MO, USA) supplemented with hemin (5 μg/mL), menadione (1 μg/mL), and 5% defibrinated horse blood. Incubation was performed in an anaerobic chamber under an atmosphere containing 5–10% H_2_, 10% CO_2_, and 80–85% N_2_ for 72 h at 37 °C.

#### 3.6.2. Minimal Inhibitory Concentration (MIC)

The minimal inhibitory concentration (MIC) is the lowest concentration of an agent capable of inhibiting microbial growth. The microdilution method was employed to determine MIC values in triplicate using 96-well microplates, following the standardized methodology of the Clinical and Laboratory Standards Institute, with modifications using resazurin as a revealing agent [55]. The compounds were dissolved in dimethyl sulfoxide (DMSO) at 1.0 mg mL^−1^, followed by dilution in broth to achieve concentrations ranging from 0.195 to 400.0 µg mL^−1^. For periodontopathogens, the inoculum was adjusted to achieve concentrations of 1 × 10^6^ CFU mL^−1^, and for cariogenic bacteria, the inoculum was adjusted to a concentration of 5 × 10^5^ CFU mL^−1^. DMSO 5% (*v*/*v*) was used as the negative control, and chlorhexidine (Sigma) was used as the positive control. The microplates were incubated under appropriate conditions for each bacterium, as described above. After incubation, 30 μL of a 0.02% aqueous resazurin (Sigma) solution was added to each well [21,56,57]. Resazurin acts as an oxidation-reduction indicator, facilitating real-time assessment of microbial viability. In this context, the blue color indicated the absence of microbial viability, whereas the red color signified the presence of microbial viability, thereby determining the value of MIC. The sample evaluated was considered bactericidal when it exhibited the same value in both the MIC and MBC assays [58]. All assays were performed in triplicate.

### 3.7. Molecular Docking

#### 3.7.1. Setup of the Systems

The docking studies presented below were performed using the AutoDock4 version (v4.2.6) algorithm [59] and using the carbazate ligands (HL^1^, HL^2^ and HL^3^) and their Zn(II) complexes (1–3), as well the antibacterial drug chlorhexidine (CHL) as the positive control used in the experimental antibacterial activity study. For the targets, two different bioreceptors were considered to perform the docking study, the ATP-binding cassette transporter peptidase domain (PEP) of the cariogenic bacteria *S. mutans* (PDB code 5XE9) [30] and the prolyl tripeptidyl aminopeptidase (PTP39) from *P. gingivalis* anaerobic bacteria (PDB code 2EEP) [31].

The PEP enzyme of the *S. mutans* bacteria had its crystal structure complexed with the carbamate [(1~{S},2~{R},4~{S},5~{R})-5-[5-(4-methoxyphenyl)-2-methyl-pyrazol-3-yl]-1-azabicyclo[2.2.2]octan-2-yl]methyl~{*N*}-propylcarbamate (CAB), an inhibitor; this structure was deposited in the Protein Data Bank (PDB) under the code 5XE9, with the resolution at 3.10 Å. The PTP39 enzyme of the *P. gingivalis* crystal structure complexed with a small inhibitor, the [(2R)-1-(L-alanyl-L-isoleucyl)pyrrolidin-2-YL] boronic acid (BOR), was deposited in the PDB under the code 2EEP, with resolution at 2.20 Å. All docking results and images were visualized and generated using the BioVia Discovery Studio [60].

#### 3.7.2. Protocol of Docking and Validation

The docking protocol to study the interaction between the ligands and the Gram-positive (*S. mutans*) and the Gram-negative (*P. gingivalis*) bacteria enzymes cited above was established following the same strategy. Two redocking studies were performed to validate the docking protocol used in this work. Those docking studies were performed using the inhibitors CAB and BOR complexed with the *S. mutans* and *P. gingivalis*, respectively. For redocking studies, we considered all enzyme residues in rigid constraint, and the rotational bonds of small molecules were assumed to be flexible. The crystal structures of the enzymes were properly prepared, previously using the PropKa 3.5 [61] protocol to set the adequate protonation state for the critical residues such as GLU, ASP, and HIS. All molecules different from amino acid residues were eliminated from the crystal structures of the enzymes, followed by the hydrogenation of the enzymes, performed using the internal AutoDock4 setup, considering only the polar hydrogens for each residue. The charge balance of the enzyme residues was performed using the Kollman charges implemented in AutoDock4. The grid box parameters to the PEP-CAR system consisted of a box with 3D dimensions at 60 × 66 × 66 Å^3^, centered in the x, y, and z coordinates at 21.403, −5575, and 13.574. For the PTP39–BOR complex, the grid box was a 3D box with 66 × 42 × 64 Å^3^, centered in the x, y, and z coordinates at −7.076, 44.604, and 64.932. For both systems, the spacing point parameter was 0.375 Å. The docking search parameters chosen were the Lamarckian Genetic Algorithm. The setup was designed to select 150 runs, generating 300 individuals for the population size, using a large number of energy evaluations out of 25 million. The RMSDs of the rigid–flexible docking studies found were 0.471 Å (to the complex PEP–CAR) and 0.595 Å (to the complex PTP39–BOR). This result presented a low geometry variation, under 2 Å, validating the docking protocols to be used with the other ligands synthesized in our group. The main interactions and binding energies, as well as the good overlapping agreement between the crystal structures of the complexes and the small in silico-docked inhibitor, are shown in Figure 4, Figure 5, Figure 6 and Figure 7.

## 4. Conclusions

Carbazate ligands and their novel Zn(II) complexes were successfully synthesized. The compounds were characterized by spectroscopic and physicochemical methods, and the complexes were also suitable for single-crystal X-ray analysis. The results indicated the formation of Zn(II) complexes where the ligand is coordinated by the *NNO*-donor system, with a distorted square-base pyramid geometry for (1) and octahedral geometry for (2–3). The complexes (1–3) and the free carbazate ligands were tested for their biological activities against different strains of cariogenic and anaerobic bacteria that cause periodontal infection. Complex (3) shows the most promising results of the synthesized compounds. A significant increase in biological activity was observed when the ligand was coordinated with the metal center. The molecular docking study corroborated the biological experimental essay about the inhibitory activity of the carbazate ligands and their Zn(II) complexes, revealing the strong ability of complex (3) to mimic the profile of interactions while comparing with known inhibitors. Furthermore, the HL^3^ ligand performed well during in silico modeling.

## Figures and Tables

**Table 1:** Selected bond lengths (Å) and angles (°) for the Zn(II) complexes (1–3).

Bond Lengths (Å)				Bond Angles (°)			
	(1)	(2)	(3)		(1)	(2)	(3)
Zn1–N1	2.171 (2)	2.185 (4)	2.160 (3)	N1–Zn1–N2	75.74 (7)	75.55 (2)	76.46 (2)
Zn1–N2	2.051 (2)	2.051 (3)	2.049 (3)	N1–Zn1–O1	151.55 (7)	148.72 (2)	151.01 (2)
Zn1–N4	-	2.162 (4)	-	N1–Zn1–N5	99.75 (8)	104.98 (2)	-
Zn1–N5	-	2.054 (3)	-	N1–Zn1–O3	99.60 (7)	94.37 (2)	-
Zn1–O1	2.137 (2)	2.186 (3)	2.151 (3)	N2–Zn1–O3	148.20 (7)	99.26 (2)	-
Zn1–O3	1.956 (2)	2.228 (3)	-	N2–Zn1–N5	-	172.79 (4)	-
Zn1–O5	2.007 (2)	-	-	O1–Zn1–O3	104.14 (6)	96.42 (2)	-
C8–O1	1.238 (3)	1.235 (4)	1.248 (4)	O3–Zn1–N5	-	73.54 (2)	-
C8–O2	1.343 (3)	1.350 (5)	1.252 (9)	N2–Zn1–N2^i^	-	-	173.29 (3)
N2–C6	1.286 (3)	1.283 (5)	1.297 (5)	C16–N5–N6	-	119.30 (4)	-
N2–N3	1.370 (3)	1.370 (4)	1.367 (4)	N3–C8–O2	110.51 (2)	110.10 (4)	113.30 (4)
N3–C8	1.348 (3)	1.334 (5)	1.336 (5)	C6–N2–N3	121.13 (2)	119.00 (4)	120.70 (3)

**Table 2:** The antibacterial activity determined for the free carbazate ligands and their Zn(II) complexes (1–3), expressed as MIC in μg mL^−1^.

Cariogenic Bacterium										
Compound	*S. mutans*	*S. sobrinus*		*S. oralis*		*S. sanguinis*		*S. salivarius*		*L. paracasei*
HL^1^	>400	>400		>400		>400		>400		>400
HL^2^	400	400		>400		400		>400		>400
HL^3^	12.5	25		50		25		100		100
(1)	200	200		400		200		400		400
(2)	100	100		200		200		200		200
(3)	12.5	12.5		25		25		25		100
Chlorhexidine	0.461	0.461		7.375		0.922		0.922		0.922
Anaerobic Bacterium										
Compound	*A. naeslundii*		*P. anaerobius*		*V. parvula*		*P. gingivalis*		*F. nucleatum*	
HL^1^	>400		>400		400		400		>400	
HL^2^	>400		100		400		200		>400	
HL^3^	100		50		100		50		200	
(1)	200		200		400		100		200	
(2)	200		100		100		50		400	
(3)	25		12.5		100		100		50	
Chlorhexidine	1.844		0.230		0.922		0.922		1.844	

**Table 3:** Docking energy of binding (E_bind_/kcal·mol^−1^), theoretical constant of inhibition (Ki_theo_/mM–micromolar), and main interactions (distance in Å) among the three carbazate ligands and their Zn(II) complexes (1–3), chlorhexidine, CAB, and CABr (redocking).

Carcinogenic Bacteria-*S. mutans* PEP Enzyme									
Residue	CAB	CABr	CHL	HL^1^	HL^2^	HL^3^	(1)	(2)	(3)
ARG66	4.45	5.47	4.99	4.83	4.97	3.66	3.59	5.48	4.84/1.75
ALA70	3.85	3.70	4.06	--	--	--	>6.00	4.43	--
ASP71	3.27	1.99	2.44	--	--	--	>6.00	--	--
LEU74	5.26	4.58	3.14	--	5.01	--	5.18	>6.00	5.50
TYR77	5.45	5.44	--	5.18	4.06	4.96	>6.00	4.96	3.15
Ki_the_	--	9.87	6.57	379.81	337.85	9.07	248.90	80.82	9.25
E_bind_	--	−6.83	−7.07	−4.67	−4.74	−6.88	−4.92	−5.58	−6.87

**Table 4:** Docking energy of binding (E_bind_/kcal·mol^−1^), theoretical constant of inhibition (Ki_theo_/mM–micromolar), and main interactions (distances in Å) between the free carbazate ligands and their Zn(II) complexes (1–3), chlorhexidine and BOR and BORr (redocking).

Anaerobic Bacteria–*P. gingivalis* PTP39 Enzyme									
Residue	BOR	BORr	CHL	HL^1^	HL^2^	HL^3^	(1)	(2)	(3)
SER603	1.76	2.45	3.30	>6.00	2.22	1.75	--	2.72	3.50
TYR518	2.65	1.69	1.79	>6.00	2.08	2.45	--	2.90	5.41
TYR635	2.63	1.97	--	1.83	3.07	4.88	2.08	5.08	>6.00
GLU205	2.62	2.78	--	3.59	--	--	5.12	--	>4.00
GLU636	2.73	2.71	4.59	--	--	--	4.94	--	--
HIS522	4.39	4.09	2.44	--	--	--	--	--	5.33
GLN203	2.77	2.47	2.01	1.93	--	--	1.94	--	>4.00
TYR604	2.96	2.30	--	--	5.52	3.31	--	--	--
TYR639	4.76	4.12	--	3.97	4.82	*4.79*	4.09	4.24	4.23
E_bind_	--	−5.83	−9.43	−4.21	−3.99	−5.33	−5.00	−6.39	−6.62
Ki_theo_		53.68	0.12	815.98	1200.0	124.53	217.33	20.86	13.97

## Data Availability

The datasets generated for this study can be found in the Supplementary Materials.

## Supplementary Materials

The following supporting information can be downloaded at https://www.mdpi.com/article/10.3390/molecules30132822/s1. Supplementary crystallographic data for the structures in this work were deposited at the Cambridge Crystallographic Data Centre, CCDC 2354507-2354509. Copies of the available material can be obtained free of charge by application to the Director, CCDC, 12 Union Road, Cambridge CH2 1EZ, UK (fax: +44 1223 336033; E-mail: deposit@ccdc.cam.ac.uk or http://www.ccdc.cam.ac.uk (Accessed on: 17 June 2025). Supplementary Tables, Figures, NMR, and IR spectra are as detailed in the text (PDF). Crystallographic data are in CIF files.
