# Supplementary material for: Exploring the Structural Design, Antibacterial Activity, and Molecular Docking of Newly Synthesized Zn(II) Complexes with NNO-Donor Carbazate Ligands
Source: Molecules. 2025 Jun 30;30(13):2822. doi: 10.3390/molecules30132822 (PMC12251245; doi:10.3390/molecules30132822)

checkCIF/PLATON report

Structure factors have been supplied for datablock(s) ccg_ed_zn7_0ma

THIS REPORT IS FOR GUIDANCE ONLY. IF USED AS PART OF A REVIEW PROCEDURE FOR PUBLICATION, IT SHOULD NOT REPLACE THE EXPERTISE OF AN EXPERIENCED CRYSTALLOGRAPHIC REFEREE.

No syntax errors found.   CIF dictionary   Interpreting this report

Datablock: ccg_ed_zn7_0ma

Bond precision: C-C = 0.0039 A   Wavelength=0.71073

Cell:	a=9.6556(12)	b=16.465(2)	c=9.1050(11)
	alpha=90	beta=104.823(2)	gamma=90
Temperature:	273 K		

	Calculated	Reported
Volume	1399.3(3)	1399.3(3)
Space group	P 21/c	P 1 21/c 1
Hall group	-P 2ybc	-P 2ybc
Moiety formula	C11 H15 N3 O5 Zn	C11 H15 N3 O5 Zn
Sum formula	C11 H15 N3 O5 Zn	C11 H15 N3 O5 Zn
Mr	334.65	334.63
Dx,g cm-3	1.589	1.588
Z	4	4
Mu (mm-1)	1.777	1.777
F000	688.0	688.0
F000'	689.42	
h,k,lmax	12,20,11	12,20,11
Nref	2884	2878
Tmin,Tmax	0.450,0.654	0.614,0.745
Tmin'	0.435	

Correction method= # Reported T Limits: Tmin=0.614 Tmax=0.745
AbsCorr = MULTI-SCAN

Data completeness= 0.998   Theta(max)= 26.438

R(reflections)= 0.0292(2301)   wR2(reflections)= 0.0749(2878)

S = 1.066   Npar= 192

The following ALERTS were generated. Each ALERT has the format
test-name_ALERT_alert-type_alert-level.
Click on the hyperlinks for more details of the test.

Alert level G

```
PLAT002_ALERT_2_G Number of Distance or Angle Restraints on AtSite          3 Note
PLAT176_ALERT_4_G The CIF-Embedded .res File Contains SADI Records          1 Report
PLAT199_ALERT_1_G Reported _cell_measurement_temperature ..... (K)        273 Check
PLAT200_ALERT_1_G Reported   _diffrn_ambient_temperature ..... (K)        273 Check
PLAT480_ALERT_4_G Long H...A H-Bond Reported H4       ..O4       .       2.62 Ang.
PLAT794_ALERT_5_G Tentative Bond Valency for Zn1       (II)      .       1.96 Info
PLAT860_ALERT_3_G Number of Least-Squares Restraints ............           1 Note
PLAT912_ALERT_4_G Missing # of FCF Reflections Above STh/L=  0.600          5 Note
PLAT969_ALERT_5_G The 'Henn et al.' R-Factor-gap value ...........      2.344 Note
              Predicted wR2: Based on SigI**2  3.19 or SHELX Weight  7.02
PLAT978_ALERT_2_G Number C-C Bonds with Positive Residual Density.          1 Info
```

0 **ALERT level A** = Most likely a serious problem - resolve or explain
0 **ALERT level B** = A potentially serious problem, consider carefully
0 **ALERT level C** = Check. Ensure it is not caused by an omission or oversight
10 **ALERT level G** = General information/check it is not something unexpected

2 ALERT type 1 CIF construction/syntax error, inconsistent or missing data
2 ALERT type 2 Indicator that the structure model may be wrong or deficient
1 ALERT type 3 Indicator that the structure quality may be low
3 ALERT type 4 Improvement, methodology, query or suggestion
2 ALERT type 5 Informative message, check

It is advisable to attempt to resolve as many as possible of the alerts in all categories. Often the minor alerts point to easily fixed oversights, errors and omissions in your CIF or refinement strategy, so attention to these fine details can be worthwhile. In order to resolve some of the more serious problems it may be necessary to carry out additional measurements or structure refinements. However, the purpose of your study may justify the reported deviations and the more serious of these should normally be commented upon in the discussion or experimental section of a paper or in the "special_details" fields of the CIF. checkCIF was carefully designed to identify outliers and unusual parameters, but every test has its limitations and alerts that are not important in a particular case may appear. Conversely, the absence of alerts does not guarantee there are no aspects of the results needing attention. It is up to the individual to critically assess their own results and, if necessary, seek expert advice.

Publication of your CIF in IUCr journals

A basic structural check has been run on your CIF. These basic checks will be run on all CIFs submitted for publication in IUCr journals (*Acta Crystallographica*, *Journal of Applied Crystallography*, *Journal of Synchrotron Radiation*); however, if you intend to submit to *Acta Crystallographica Section C* or *E* or *IUCrData*, you should make sure that full publication checks are run on the final version of your CIF prior to submission.

Publication of your CIF in other journals

Please refer to the *Notes for Authors* of the relevant journal for any special instructions relating to CIF submission.

PLATON version of 02/02/2025; check.def file version of 02/02/2025

Datablock ccg_ed_zn7_0ma - ellipsoid plot

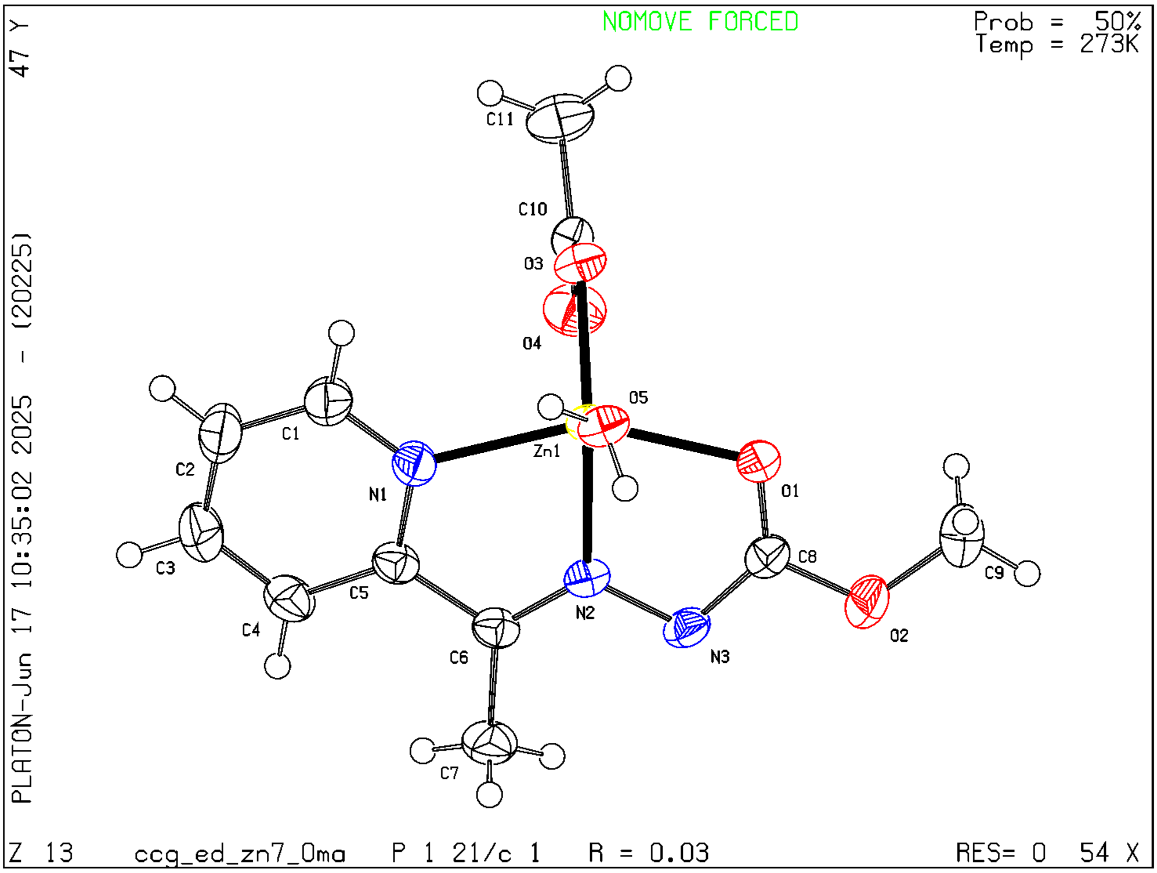

Supplement: Supplementary file 1 [file molecules-30-02822-s001.zip › Checkcif(1).pdf]
