# Supplementary material for: Exploring the Structural Design, Antibacterial Activity, and Molecular Docking of Newly Synthesized Zn(II) Complexes with NNO-Donor Carbazate Ligands
Source: Molecules. 2025 Jun 30;30(13):2822. doi: 10.3390/molecules30132822 (PMC12251245; doi:10.3390/molecules30132822)

checkCIF/PLATON report

Structure factors have been supplied for datablock(s) ccg_ed_zn6_0ma

THIS REPORT IS FOR GUIDANCE ONLY. IF USED AS PART OF A REVIEW PROCEDURE FOR PUBLICATION, IT SHOULD NOT REPLACE THE EXPERTISE OF AN EXPERIENCED CRYSTALLOGRAPHIC REFEREE.

No syntax errors found. CIF dictionary Interpreting this report

Datablock: ccg_ed_zn6_0ma

Bond precision:	C-C = 0.0067 A		Wavelength=0.71073
Cell:	a=10.178(3)	b=21.459(5)	c=10.155(3)
	alpha=90	beta=90.435(7)	gamma=90
Temperature:	296 K		

	Calculated	Reported
Volume	2217.9(11)	2217.8(10)
Space group	P 21/c	P 1 21/c 1
Hall group	-P 2ybc	-P 2ybc
Moiety formula	C20 H24 N6 O4 Zn	C20 H24 N6 O4 Zn
Sum formula	C20 H24 N6 O4 Zn	C20 H24 N6 O4 Zn
Mr	477.84	477.82
Dx,g cm-3	1.431	1.431
Z	4	4
Mu (mm-1)	1.146	1.146
F000	992.0	992.0
F000’	993.47	
h,k,lmax	12,25,12	12,25,12
Nref	3893	3893
Tmin,Tmax	0.577,0.830	0.584,0.745
Tmin’	0.565	

Correction method= # Reported T Limits: Tmin=0.584 Tmax=0.745
AbsCorr = MULTI-SCAN

Data completeness= 1.000 Theta(max)= 24.997

R(reflections)= 0.0528(2236) wR2(reflections)= 0.1220(3893)

S = 1.015 Npar= 284

The following ALERTS were generated. Each ALERT has the format
test-name_ALERT_alert-type_alert-level.
Click on the hyperlinks for more details of the test.

Alert level C

```
PLAT341_ALERT_3_C Low Bond Precision on  C-C Bonds ...............    0.00671 Ang.
PLAT905_ALERT_3_C Negative K value in the Analysis of Variance ...     -1.417 Report
```

Alert level G

```
PLAT794_ALERT_5_G Tentative Bond Valency for Zn1       (II)      .       1.92 Info
PLAT941_ALERT_3_G Average HKL Measurement Multiplicity ..........         3.6 Low
PLAT960_ALERT_3_G Number of Intensities with I < - 2*Sigma(I) ....          1 Check
PLAT967_ALERT_5_G Note: Two-Theta Cutoff Value in Embedded .res ..       50.0 Degree
PLAT969_ALERT_5_G The 'Henn et al.' R-Factor-gap value ..........      1.769 Note
              Predicted wR2: Based on SigI**2  6.90 or SHELX Weight 12.02
PLAT978_ALERT_2_G Number C-C Bonds with Positive Residual Density.          0 Info
```

0 **ALERT level A** = Most likely a serious problem - resolve or explain
0 **ALERT level B** = A potentially serious problem, consider carefully
2 **ALERT level C** = Check. Ensure it is not caused by an omission or oversight
6 **ALERT level G** = General information/check it is not something unexpected

0 ALERT type 1 CIF construction/syntax error, inconsistent or missing data
1 ALERT type 2 Indicator that the structure model may be wrong or deficient
4 ALERT type 3 Indicator that the structure quality may be low
0 ALERT type 4 Improvement, methodology, query or suggestion
3 ALERT type 5 Informative message, check

It is advisable to attempt to resolve as many as possible of the alerts in all categories. Often the minor alerts point to easily fixed oversights, errors and omissions in your CIF or refinement strategy, so attention to these fine details can be worthwhile. In order to resolve some of the more serious problems it may be necessary to carry out additional measurements or structure refinements. However, the purpose of your study may justify the reported deviations and the more serious of these should normally be commented upon in the discussion or experimental section of a paper or in the "special_details" fields of the CIF. checkCIF was carefully designed to identify outliers and unusual parameters, but every test has its limitations and alerts that are not important in a particular case may appear. Conversely, the absence of alerts does not guarantee there are no aspects of the results needing attention. It is up to the individual to critically assess their own results and, if necessary, seek expert advice.

Publication of your CIF in IUCr journals

A basic structural check has been run on your CIF. These basic checks will be run on all CIFs submitted for publication in IUCr journals (*Acta Crystallographica*, *Journal of Applied Crystallography*, *Journal of Synchrotron Radiation*); however, if you intend to submit to *Acta Crystallographica Section C* or *E* or *IUCrData*, you should make sure that full publication checks are run on the final version of your CIF prior to submission.

Publication of your CIF in other journals

Please refer to the *Notes for Authors* of the relevant journal for any special instructions relating to CIF submission.

PLATON version of 02/02/2025; check.def file version of 02/02/2025

Datablock ccg_ed_zn6_0ma - ellipsoid plot

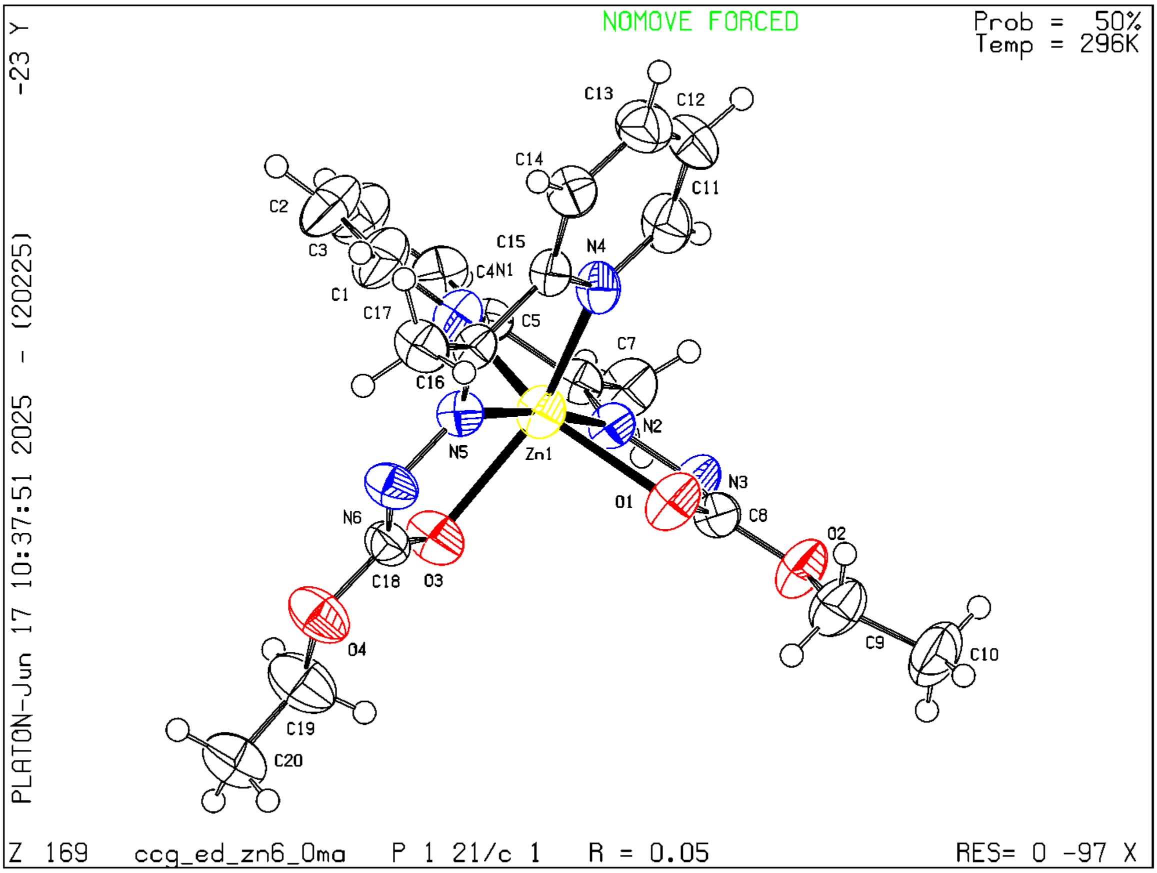

Supplement: Supplementary file 1 [file molecules-30-02822-s001.zip › Checkcif(2).pdf]
