# Supplementary material for: Exploring the Structural Design, Antibacterial Activity, and Molecular Docking of Newly Synthesized Zn(II) Complexes with NNO-Donor Carbazate Ligands
Source: Molecules. 2025 Jun 30;30(13):2822. doi: 10.3390/molecules30132822 (PMC12251245; doi:10.3390/molecules30132822)

checkCIF/PLATON report

Structure factors have been supplied for datablock(s) shelx

THIS REPORT IS FOR GUIDANCE ONLY. IF USED AS PART OF A REVIEW PROCEDURE FOR PUBLICATION, IT SHOULD NOT REPLACE THE EXPERTISE OF AN EXPERIENCED CRYSTALLOGRAPHIC REFEREE.

No syntax errors found.   CIF dictionary   Interpreting this report

Datablock: shelx

Bond precision: C-C = 0.0067 A   Wavelength=0.71073

Cell:	a=12.0841(17)	b=9.8085(13)	c=24.457(3)
	alpha=90	beta=90	gamma=90
Temperature:	296 K		

	Calculated	Reported
Volume	2898.8(7)	2898.8(7)
Space group	P b c n	P b c n
Hall group	-P 2n 2ab	-P 2n 2ab
Moiety formula	C30 H28 N6 O4 Zn	?
Sum formula	C30 H28 N6 O4 Zn	C30 H28 N6 O4 Zn
Mr	601.97	601.95
Dx,g cm-3	1.379	1.379
Z	4	4
Mu (mm-1)	0.893	0.893
F000	1248.0	1248.0
F000'	1249.56	
h,k,lmax	14,11,29	14,11,29
Nref	2559	2558
Tmin,Tmax	0.722,0.772	0.708,0.722
Tmin'	0.708	

Correction method= # Reported T Limits: Tmin=0.708 Tmax=0.722
AbsCorr = MULTI-SCAN

Data completeness= 1.000   Theta(max)= 25.000

R(reflections)= 0.0471(1538)   wR2(reflections)= 0.1197(2558)

S = 1.020   Npar= 332

The following ALERTS were generated. Each ALERT has the format
test-name_ALERT_alert-type_alert-level.
Click on the hyperlinks for more details of the test.

Alert level B

```
PLAT088_ALERT_3_B Poor Data / Parameter Ratio ...................       7.70 Note
```

Alert level C

```
ABSTY02_ALERT_1_C  An _exptl_absorpt_correction_type has been given without
            a literature citation. This should be contained in the
            _exptl_absorpt_process_details field.
            Absorption correction given as Multi-scan
PLAT230_ALERT_2_C Hirshfeld Test Diff for    O1       --C8       .        5.7 s.u.
PLAT234_ALERT_4_C Large Hirshfeld Difference O2       --C9       .       0.17 Ang.
PLAT234_ALERT_4_C Large Hirshfeld Difference O2       --C9B      .       0.20 Ang.
PLAT241_ALERT_2_C High   'MainMol' Ueq as Compared to Neighbors of         O2 Check
PLAT341_ALERT_3_C Low Bond Precision on  C-C Bonds ...............    0.00667 Ang.
PLAT906_ALERT_3_C Large K Value in the Analysis of Variance ......      7.617 Check
```

Alert level G

```
PLAT002_ALERT_2_G Number of Distance or Angle Restraints on AtSite         29 Note
PLAT003_ALERT_2_G Number of Uiso or U(i,j)  Restrained non-H-Atoms         28 Report
PLAT175_ALERT_4_G The CIF-Embedded .res File Contains SAME Records          3 Report
PLAT176_ALERT_4_G The CIF-Embedded .res File Contains SADI Records          1 Report
PLAT177_ALERT_4_G The CIF-Embedded .res File Contains DELU Records          4 Report
PLAT179_ALERT_4_G The CIF-Embedded .res File Contains CHIV Records          3 Report
PLAT186_ALERT_4_G The CIF-Embedded .res File Contains ISOR Records          1 Report
PLAT189_ALERT_3_G A Non-default SAME Restraint Value for First Par     0.0100 Report
PLAT189_ALERT_3_G A Non-default SAME Restraint Value for First Par     0.0100 Report
PLAT189_ALERT_3_G A Non-default SAME Restraint Value for First Par     0.0100 Report
PLAT191_ALERT_3_G A Non-default SADI Restraint Value has been used     0.0100 Report
PLAT301_ALERT_3_G Main Residue  Disorder ..............(Resd    1)        34% Note
PLAT794_ALERT_5_G Tentative Bond Valency for Zn1       (II)      .       2.03 Info
PLAT811_ALERT_5_G No ADDSYM Analysis: Too Many Excluded Atoms ....          ! Info
PLAT860_ALERT_3_G Number of Least-Squares Restraints .............        328 Note
PLAT883_ALERT_1_G Absent Datum for _atom_sites_solution_primary ..     Please Do !
PLAT899_ALERT_4_G SHELXL2018 is Outdated   and Succeeded by SHELXL     2019/3 Note
PLAT909_ALERT_3_G Percentage of I>2sig(I) Data at Theta(Max) Still        31% Note
PLAT933_ALERT_2_G Number of HKL-OMIT Records in Embedded .res File          1 Note
                2  0  0,
PLAT955_ALERT_1_G Reported (CIF) and Actual (FCF) Lmax Differ by .          1 Units
PLAT960_ALERT_3_G Number of Intensities with I < - 2*Sigma(I) ....          1 Check
PLAT967_ALERT_5_G Note: Two-Theta Cutoff Value in Embedded .res ..       50.0 Degree
PLAT969_ALERT_5_G The 'Henn et al.' R-Factor-gap value ..........       3.173 Note
              Predicted wR2: Based on SigI**2  3.77 or SHELX Weight 11.73
PLAT978_ALERT_2_G Number C-C Bonds with Positive Residual Density.          7 Info
```

0 **ALERT level A** = Most likely a serious problem - resolve or explain
1 **ALERT level B** = A potentially serious problem, consider carefully
7 **ALERT level C** = Check. Ensure it is not caused by an omission or oversight
24 **ALERT level G** = General information/check it is not something unexpected

```
  3 ALERT type 1 CIF construction/syntax error, inconsistent or missing data
  6 ALERT type 2 Indicator that the structure model may be wrong or deficient
 11 ALERT type 3 Indicator that the structure quality may be low
  8 ALERT type 4 Improvement, methodology, query or suggestion
  4 ALERT type 5 Informative message, check
```

It is advisable to attempt to resolve as many as possible of the alerts in all categories. Often the minor alerts point to easily fixed oversights, errors and omissions in your CIF or refinement strategy, so attention to these fine details can be worthwhile. In order to resolve some of the more serious problems it may be necessary to carry out additional measurements or structure refinements. However, the purpose of your study may justify the reported deviations and the more serious of these should normally be commented upon in the discussion or experimental section of a paper or in the "special_details" fields of the CIF. checkCIF was carefully designed to identify outliers and unusual parameters, but every test has its limitations and alerts that are not important in a particular case may appear. Conversely, the absence of alerts does not guarantee there are no aspects of the results needing attention. It is up to the individual to critically assess their own results and, if necessary, seek expert advice.

Publication of your CIF in IUCr journals

A basic structural check has been run on your CIF. These basic checks will be run on all CIFs submitted for publication in IUCr journals (*Acta Crystallographica*, *Journal of Applied Crystallography*, *Journal of Synchrotron Radiation*); however, if you intend to submit to *Acta Crystallographica Section C* or *E* or *IUCrData*, you should make sure that full publication checks are run on the final version of your CIF prior to submission.

Publication of your CIF in other journals

Please refer to the *Notes for Authors* of the relevant journal for any special instructions relating to CIF submission.

PLATON version of 02/02/2025; check.def file version of 02/02/2025

Datablock shelx - ellipsoid plot

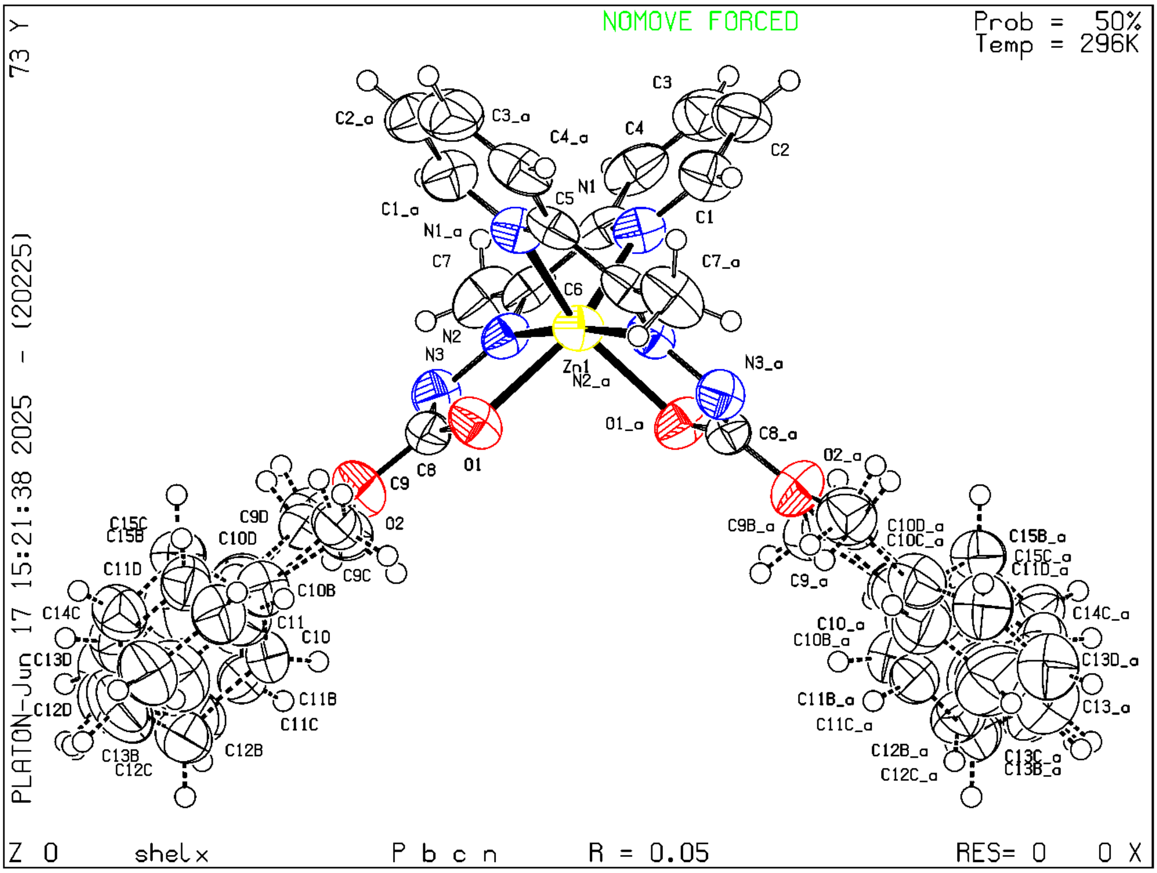

Supplement: Supplementary file 1 [file molecules-30-02822-s001.zip › Checkcif(3).pdf]
