# Supplementary material for: Exploring the Structural Design, Antibacterial Activity, and Molecular Docking of Newly Synthesized Zn(II) Complexes with NNO-Donor Carbazate Ligands
Source: Molecules. 2025 Jun 30;30(13):2822. doi: 10.3390/molecules30132822 (PMC12251245; doi:10.3390/molecules30132822)
Supplement: Supplementary file 1 [file molecules-30-02822-s001.zip › molecules-3705565-supplementary.pdf]

Exploring the Structural Design, Antibacterial Activity, and Molecular Docking of Newly Synthesized Zn(II) Complexes with *NNO*-Donor Carbazate Ligands

Supporting Information

Claudia C. Gatto [1,*], Daniel J. de Siqueira [1], Eduardo de A. Duarte [1], Érica C. M. Nascimento [2], João B. L. Martins [2], Mariana B. Santiago [3], Nagela B. S. Silva [3] and Carlos H. G. Martins [3]

[1] Laboratory of Inorganic Synthesis and Crystallography, Institute of Chemistry, University of Brasilia, Brasília 70904-970, DF, Brazil
[2] Laboratory of Computational Chemistry, Institute of Chemistry, University of Brasilia, Brasília 70904-970, DF, Brazil; ericamoreno@unb.br (É.C.M.N.); lopes@unb.br (J.B.L.M.)
[3] Laboratory of Antimicrobial Testing, Institute of Biomedical Sciences, University of Uberlândia, Campus Umuarama, Uberlândia 38405-320, MG, Brazil; carlos.martins2@ufu.br (C.H.G.M.)
* Correspondence: ccgatto@unb.br

Contents:

Figure S1: Intermolecular hydrogen interactions present in the Complex (1)……………..…2

Figure S2: Total fingerprint plots and key complex (1) interactions……………………......2

Figure S3: Total fingerprint plots and key complex (2) interactions…………………….....3

Figure S4: Total fingerprint plots and key complex (3) interactions…………………….....3

Figure S5: Infrared spectra of HL^1…………………………………………………………..4

Figure S6: Infrared spectra of HL^2…………………………………………………………..4

Figure S7: Infrared spectra of HL^3…………………………………………………………..5

Figure S8: Infrared spectra of complex (1)……………………………………………………5

Figure S9: Infrared spectra of complex (2)……………………………………………………6

Figure S10: Infrared spectra of complex (3)………………………………………………...6

Figure S11: UV-Vis spectra of HL^1 and complex (1) in MeOH, DMF, and DMSO…………7

Figure S12: UV-Vis spectra of HL^2 and complex (2) in MeOH, DMF, and DMSO…………7

Figure S13: UV-Vis spectra of HL^3 and complex (3) in MeOH, DMF, and DMSO…………7

Table S1: Absorption values of the bands observed in the electronic spectra of HL^{1-3} and (1–3)………………………………………………………………………………………8

Figure S14: ^1H NMR spectra of HL^1 in DMSO-d6……………………………………………...8

Figure S15: ^1H NMR spectra of Complex (1) in DMSO-d6…………………………………9

Figure S16: ^1H NMR spectra of HL^2 in DMSO-d6……………………………………………...9

Figure S17: ^1H NMR spectra of Complex (2) in DMSO-d6…………………………………10

Figure S18: ^1H NMR spectra of HL^3 in DMSO-d_6…………………………………………..10

Figure S19: ^1H NMR spectra of complex (3) in DMSO-d_6………………………………….11

Table S2. X-ray diffraction data collection and refinement parameters for the complexes (1–3)...……………………………………………………………………………………12

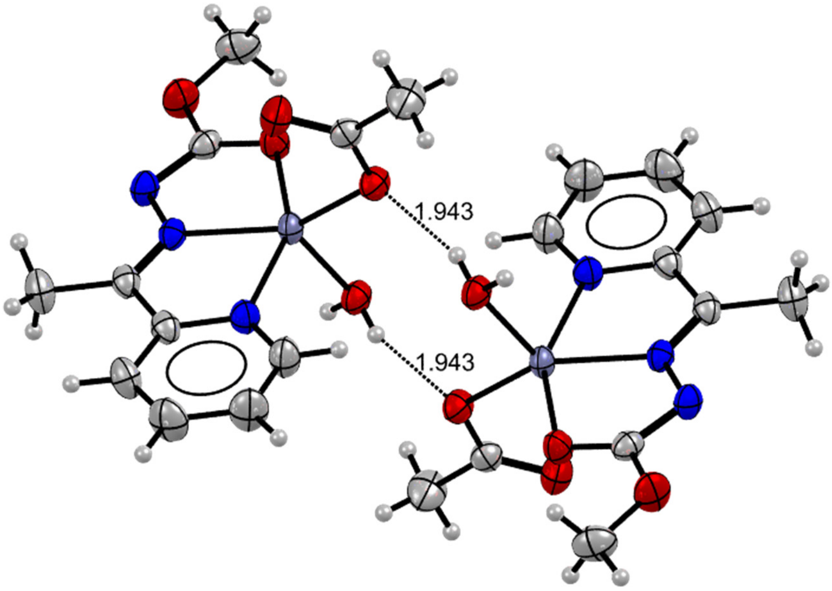

Figure S1: Intermolecular hydrogen interactions present in the Complex (1).

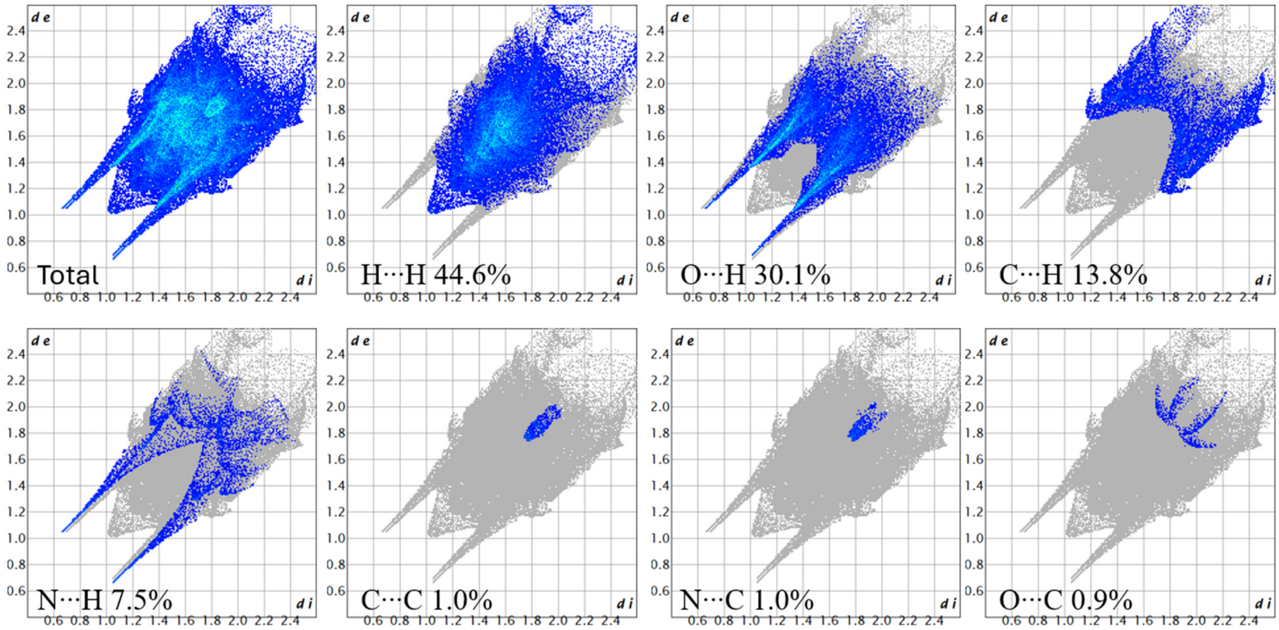

Figure S2: Total fingerprint plots and key complex (1) interactions.

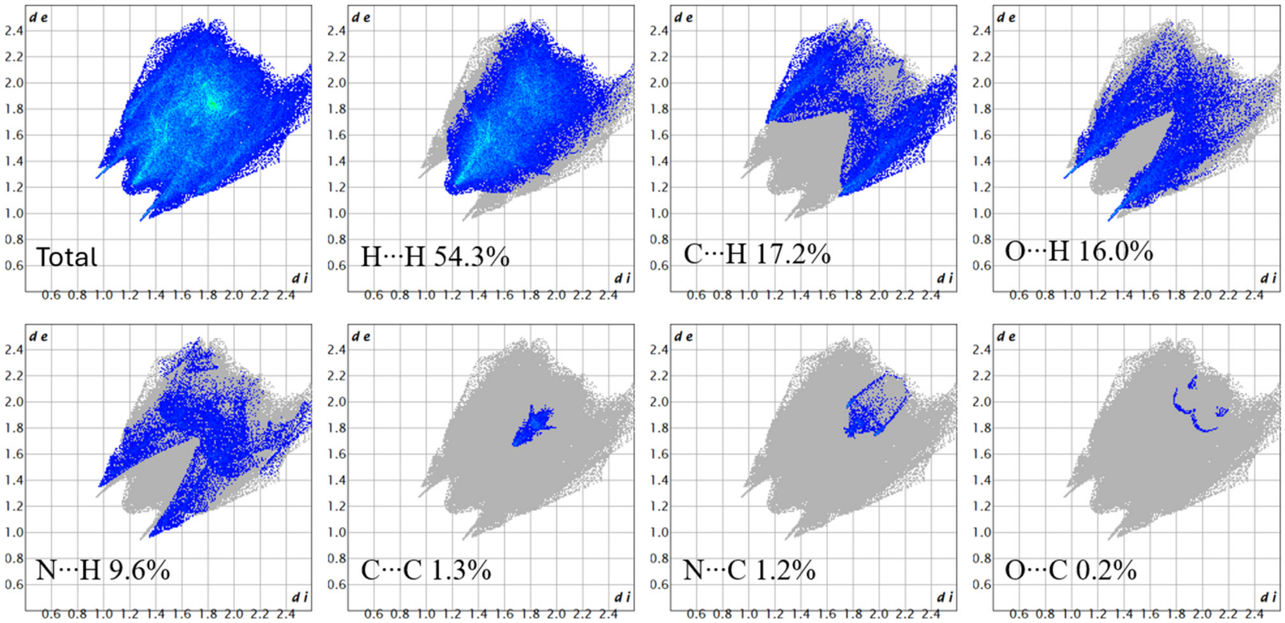

Figure S3: Total fingerprint plots and key complex (2) interactions.

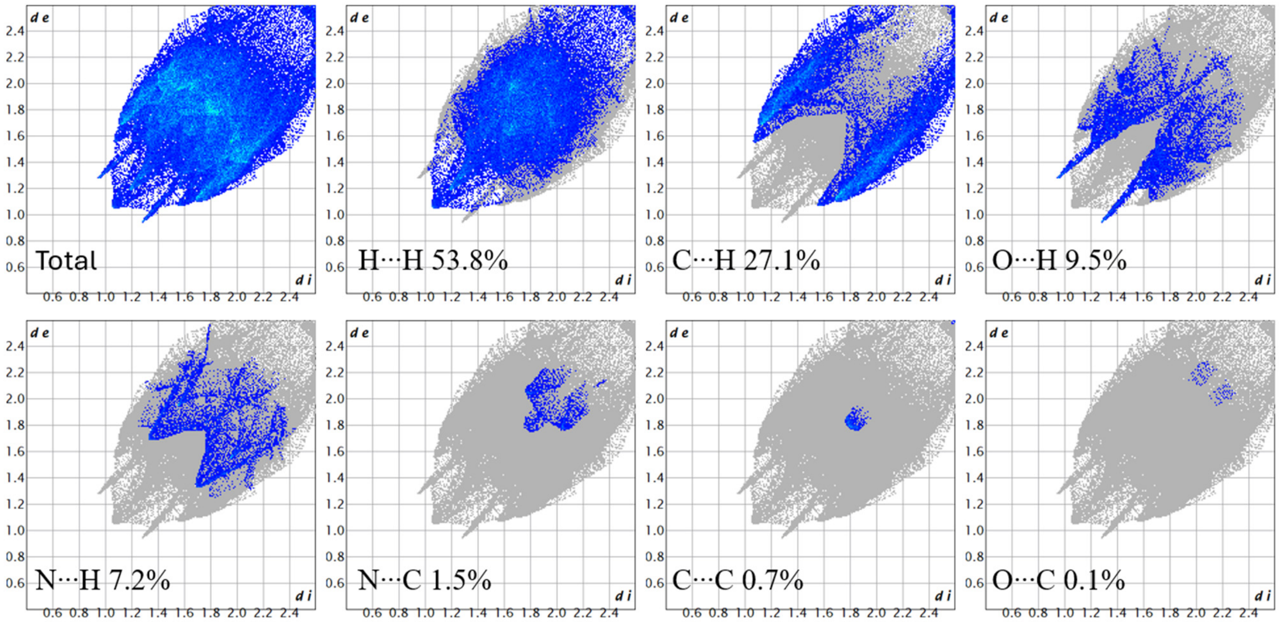

Figure S4: Total fingerprint plots and key complex (3) interactions.

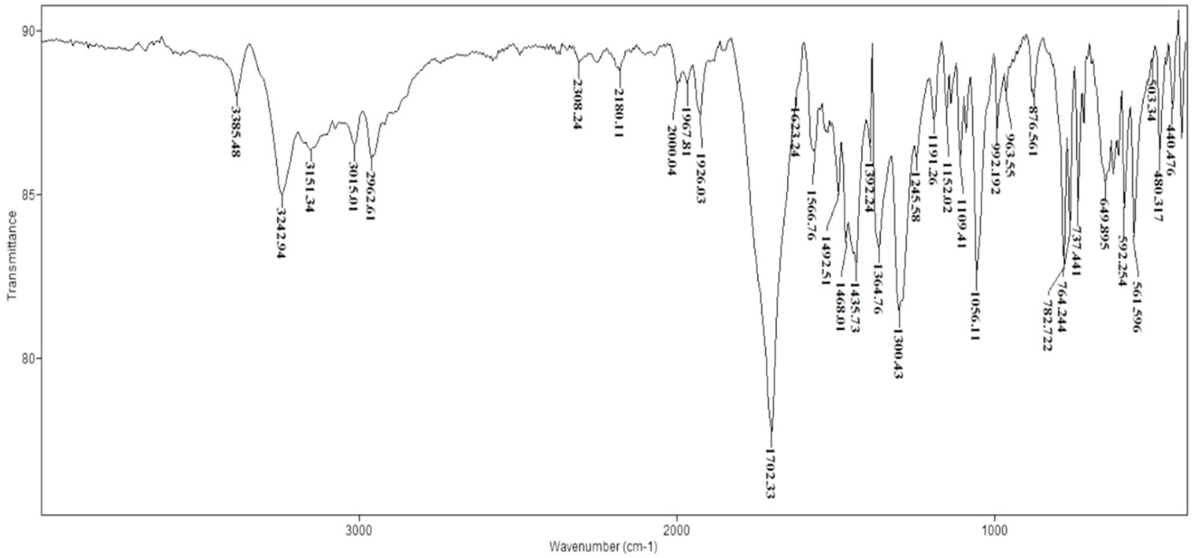

Figure S5: Infrared spectra of HL[1].

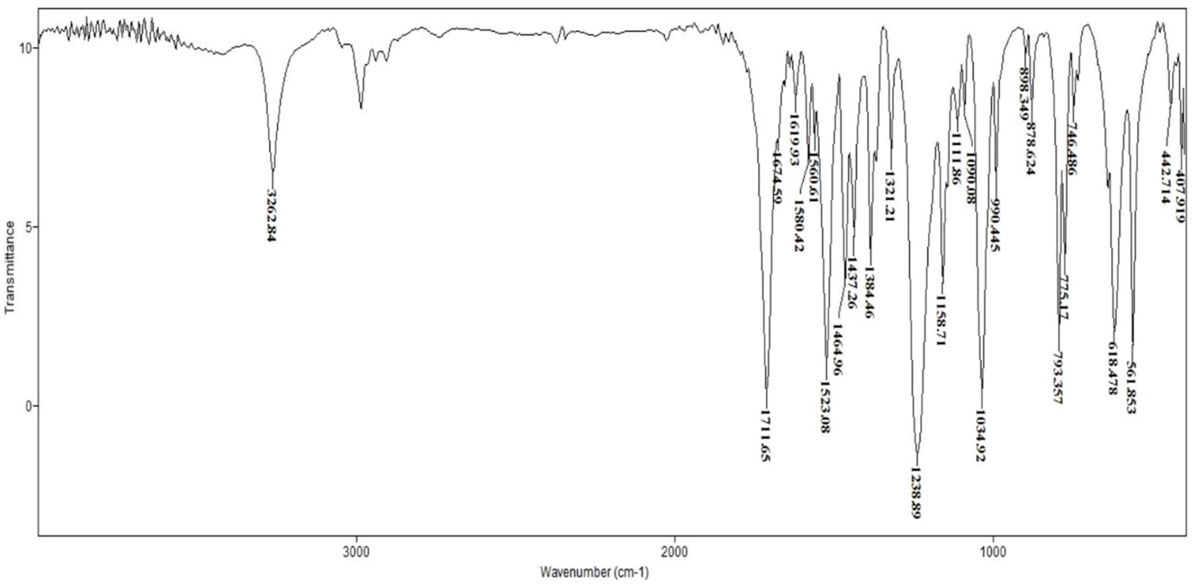

Figure S6: Infrared spectra of HL[2].

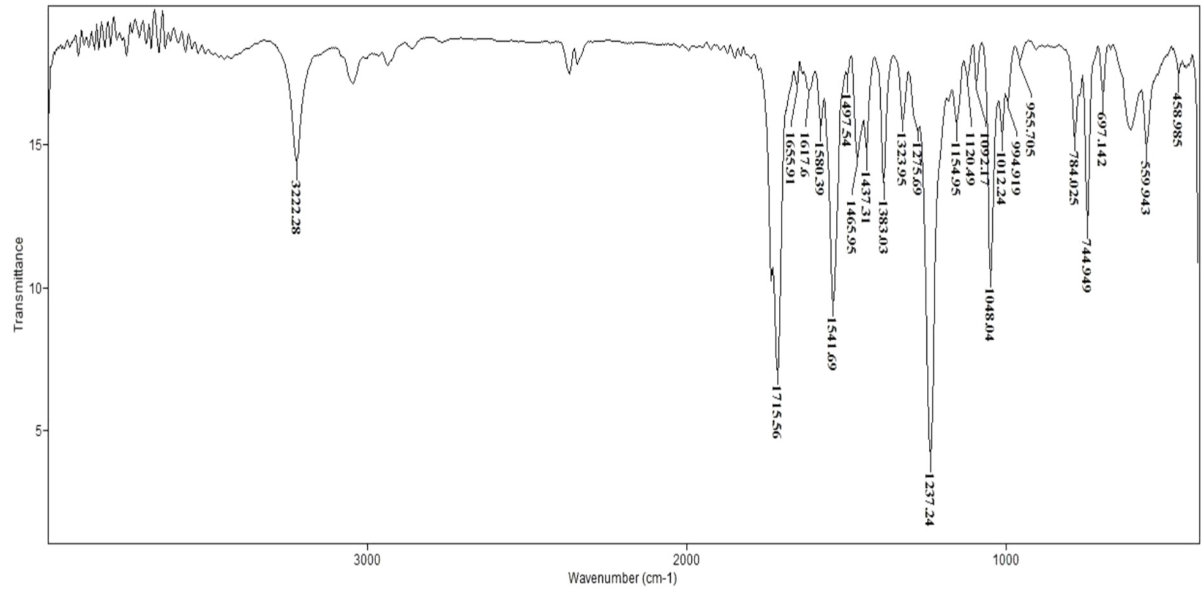

Figure S7: Infrared spectra of HL^3.

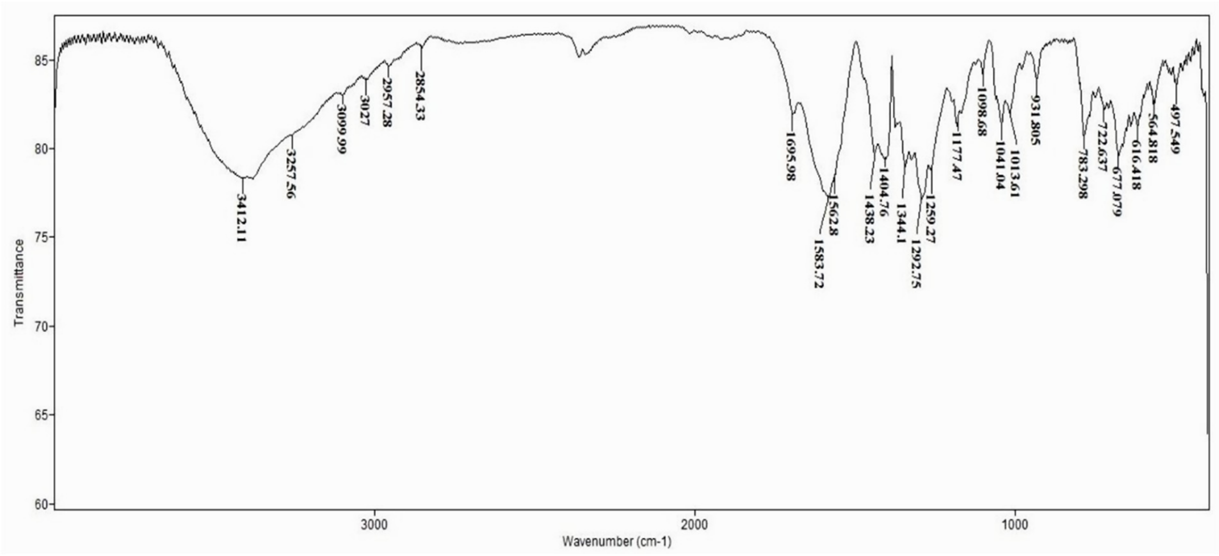

Figure S8: Infrared spectra of complex (1).

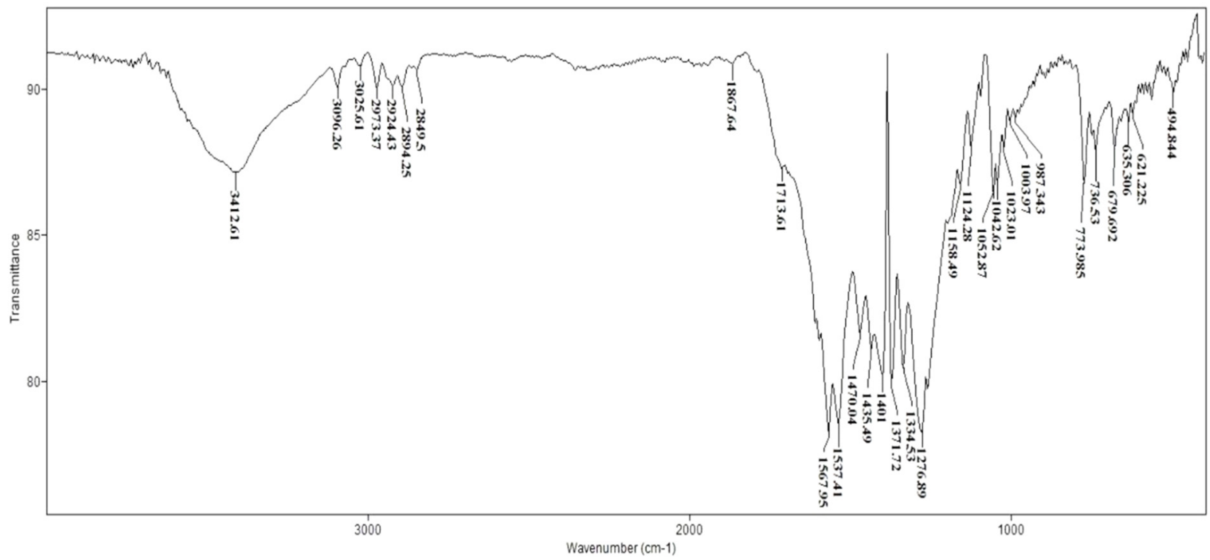

Figure S9: Infrared spectra of complex (2).

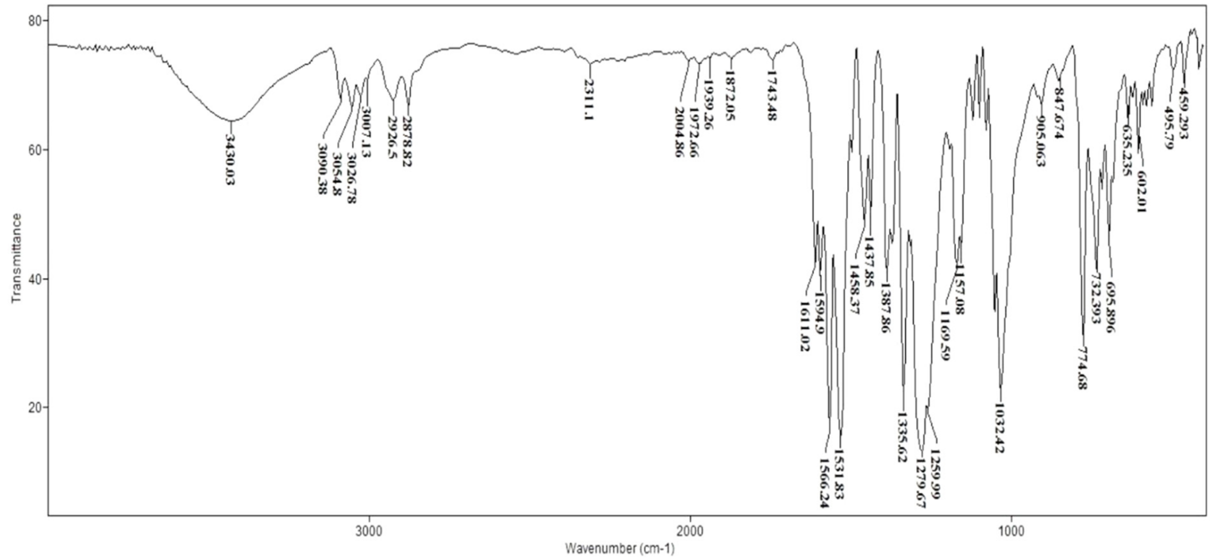

Figure S10: Infrared spectra of complex (3).

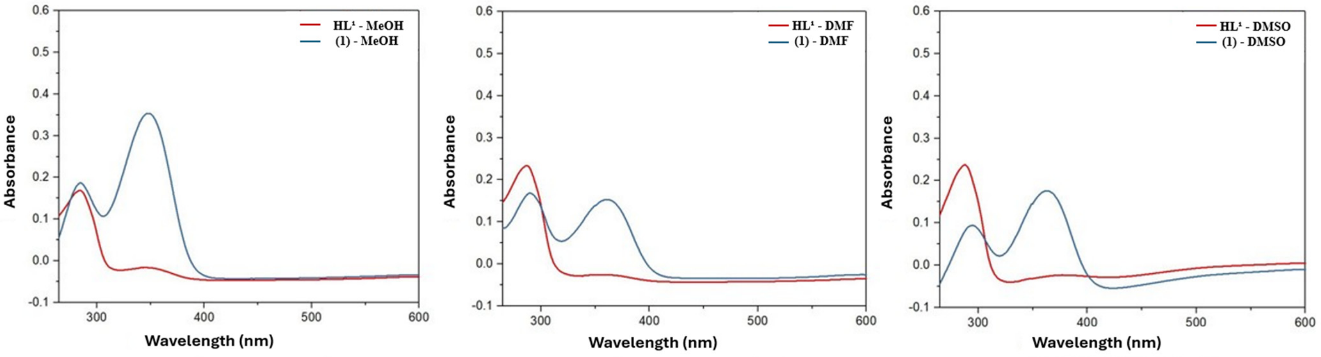

Figure S11: UV-Vis spectra of HL[1] and complex (1) in MeOH, DMF, and DMSO.

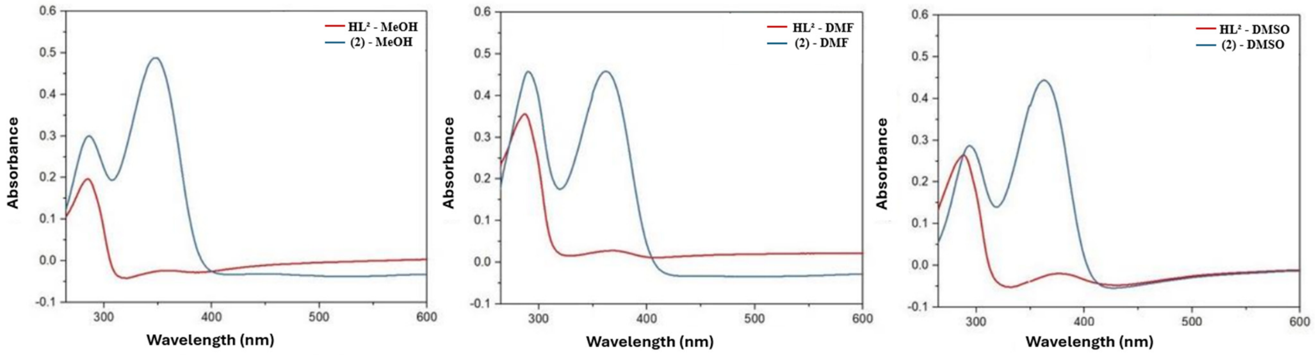

Figure S12: UV-Vis spectra of HL[2] and complex (2) in MeOH, DMF, and DMSO.

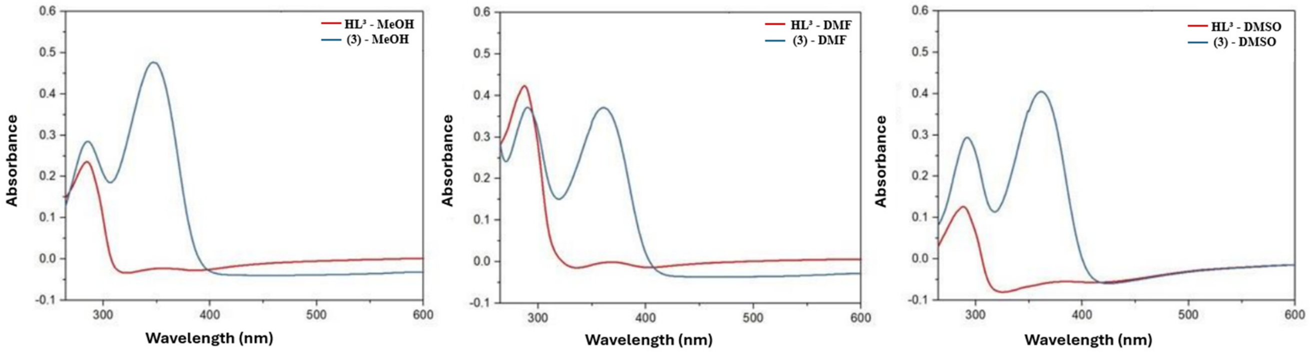

Figure S13: UV-Vis spectra of HL[3] and complex (3) in MeOH, DMF, and DMSO.

Table S1: Absorption values of the bands observed in the electronic spectra of HL^{1-3} and (1-3).

**Compound**	**π→π* Transition (nm)**			**n→π* Transition (nm)**			**LMCT Transition (nm)**		
	MeOH	DMF	DMSO	MeOH	DMF	DMSO	MeOH	DMF	DMSO
**HL^1**	284 (3.93)	287 (4.07)	288 (4.06)	347 (2.90)	356 (3.11)	377 (3.06)	-	-	-
**HL^2**	285 (3.99)	287 (4.25)	288 (4.11)	357 (3.07)	367 (3.15)	377 (2.99)	-	-	-
**HL^3**	285 (4.07)	288 (4.33)	288 (2.80)	355 (3.05)	368 (1.68)	383 (3.40)	-	-	-
**(1)**	285 (3.97)	290 (3.92)	295 (3.65)	-	-	-	349 (4.25)	361 (3.88)	363 (3.94)
**(2)**	286 (4.18)	290 (4.36)	294 (4.15)	-	-	-	348 (4.39)	362 (4.36)	363 (4.35)
**(3)**	285 (4.15)	290 (4.27)	292 (4.17)	-	-	-	347 (4.38)	361 (4.27)	361 (4.31)

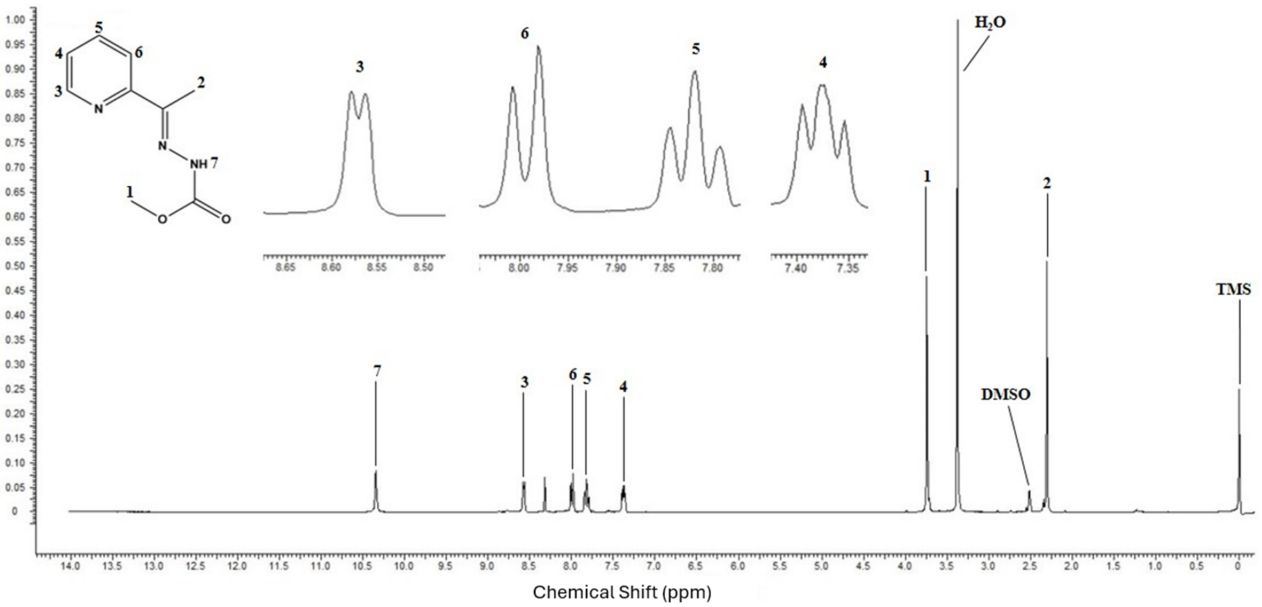

Figure S14: ^{1}H NMR spectra of HL^1 in DMSO-d_6.

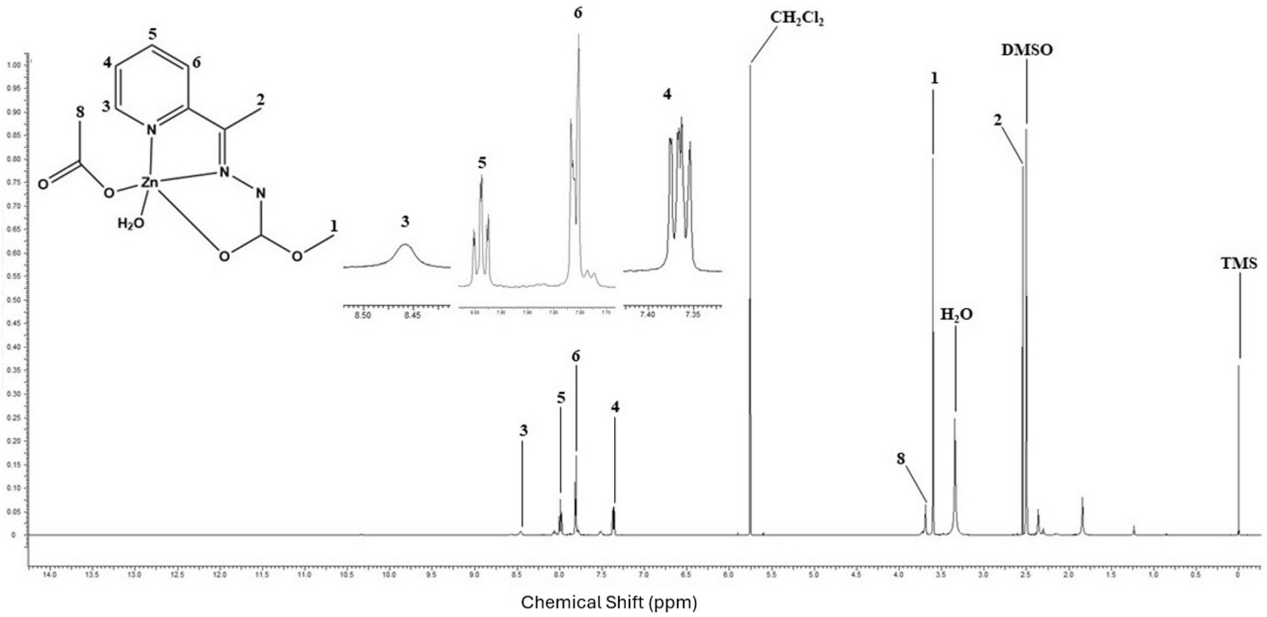

Figure S15: ^{1}H NMR spectra of Complex (1) in DMSO-d$_6$.

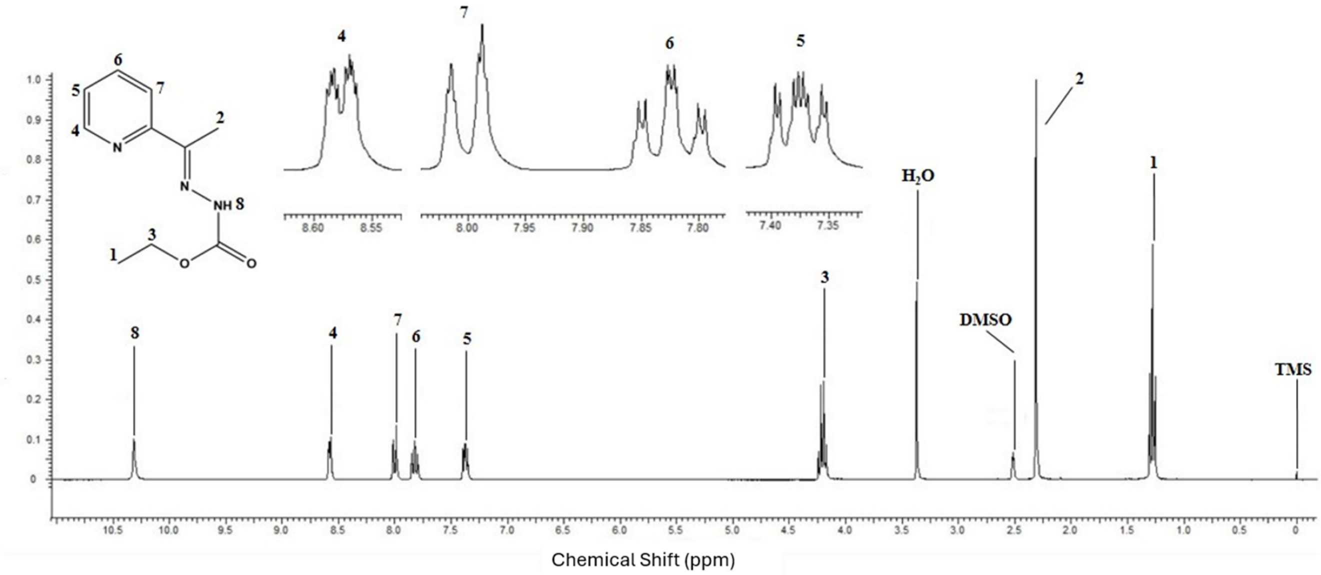

Figure S16: ^{1}H NMR spectra of HL^2 in DMSO-d$_6$.

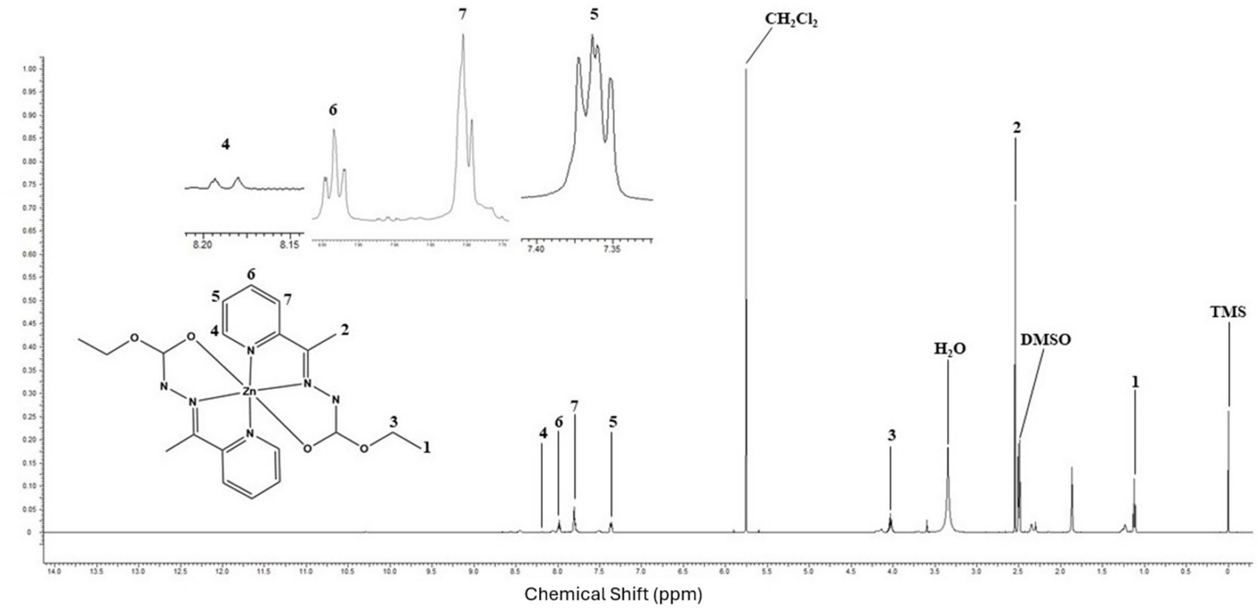

Figure S17: ^{1}H NMR spectra of Complex (2) in DMSO-d$_6$.

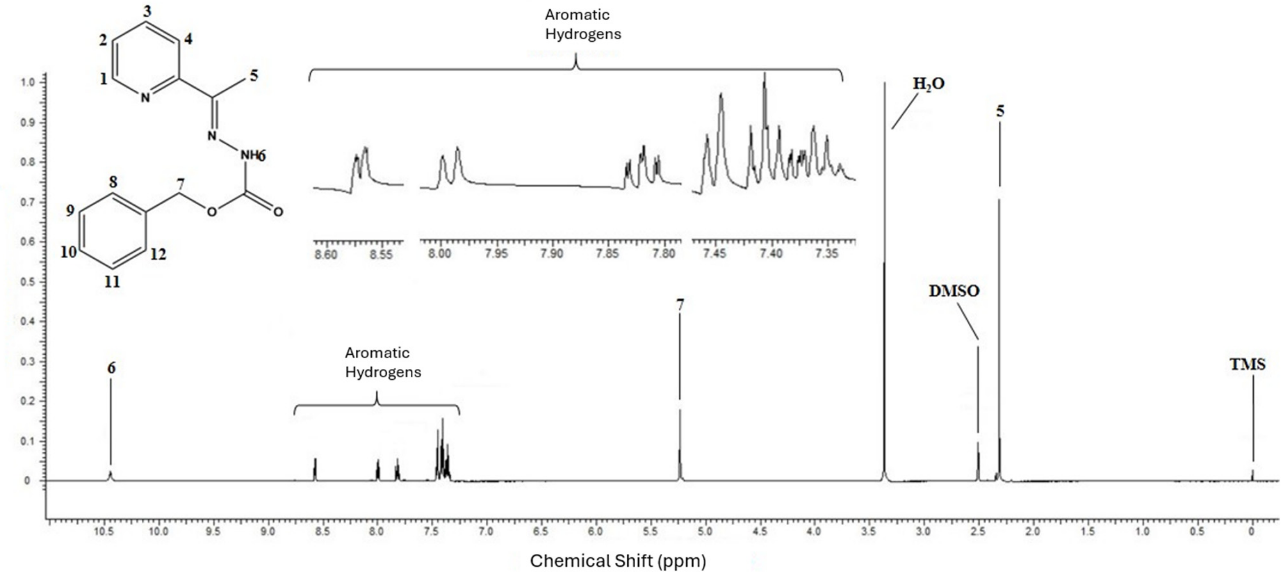

Figure S18: ^{1}H NMR spectra of HL^3 in DMSO-d$_6$.

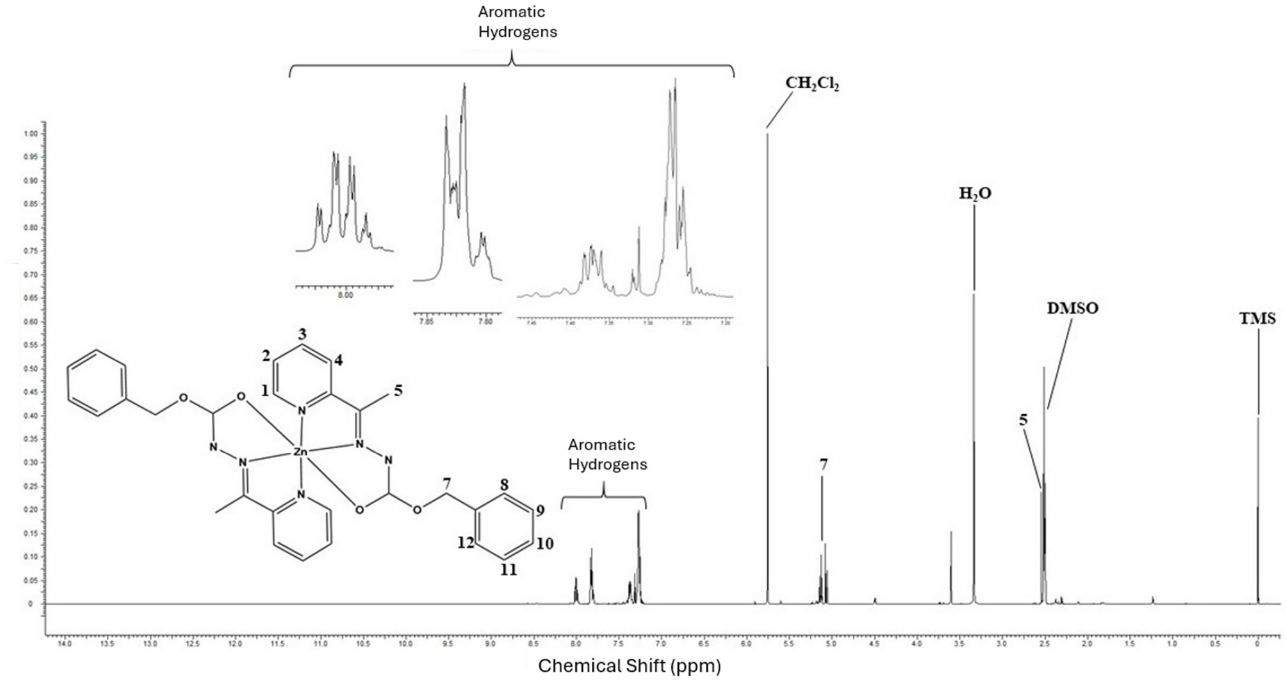

Figure S19: ^{1}H NMR spectra of complex (3) in DMSO-d$_6$.

Table S2. X-ray diffraction data collection and refinement parameters for the complexes (1–3).

	**(1)**	**(2)**	**(3)**
Chemical formula	$C_{11}H_{15}N_3O_5Zn$	$C_{20}H_{24}N_6O_4Zn$	$C_{30}H_{28}N_6O_4Zn$
M (g mol^{-1})	334.63	477.82	601.95
Crystal system	Monoclinic	Monoclinic	Orthorhombic
Space group	*P2₁/c*	*P2₁/c*	*Pbcn*
Unit cell			
a (Å)	9.656(12)	10.178(3)	12.084(17)
b (Å)	16.465(2)	21.459(5)	9.809(13)
c (Å)	9.105(11)	10.155(3)	24.457(3)
β (°)	104.823(2)	90.435	90
V (Å^3)	1399.3(3)	2217.8(10)	2898.8(7)
Z	4	4	4
Density (Mg/^{m3})	1.588	1.431	1.379
Index ranges	$-10 \leq h \leq 12$<br>$-20 \leq k \leq 20$<br>$-11 \leq l \leq 11$	$-12 \leq h \leq 12$<br>$-16 \leq k \leq 25$<br>$-9 \leq l \leq 12$	$-6 \leq h \leq 14$<br>$-11 \leq k \leq 11$<br>$-29 \leq l \leq 27$
Absorption coefficient /mm^{-1}	1.777	1.146	0.893
Absorption correction	multi-scan	multi-scan	multi-scan
Max/min transmission	0.677/0.495	0.836/0.606	0.975/0.994
Measured reflections	14781	14091	13356
Independent reflections / Rint	2878/0.043	3893/0.080	2558/0.054
Refined parameters	192	284	332
R_1 (F)	0.029	0.053	0.047
wR_2 (F^2) ($I > 2\sigma(I)$)	0.069	0.099	0.100
GooF	1.065	1.015	1.020
Largest diff. peak and hole (eÅ^{-3})	0.256 and -0.276	0.298 and -0.508	0.263 and -0.303
CCDC number	2354507	2354508	2354509
